# Models meet data: Challenges and opportunities in implementing land management in Earth system models

**DOI:** 10.1111/gcb.13988

**Published:** 2017-12-13

**Authors:** Julia Pongratz, Han Dolman, Axel Don, Karl‐Heinz Erb, Richard Fuchs, Martin Herold, Chris Jones, Tobias Kuemmerle, Sebastiaan Luyssaert, Patrick Meyfroidt, Kim Naudts

**Affiliations:** ^1^ Max Planck Institute for Meteorology Hamburg Germany; ^2^ Department of Earth Sciences VU University Amsterdam Amsterdam The Netherlands; ^3^ Thünen‐Institute of Climate‐Smart Agriculture Braunschweig Germany; ^4^ Institute of Social Ecology Vienna (SEC) Alpen‐Adria Universitaet Klagenfurt Wien, Graz Vienna Austria; ^5^ Geography Group, Department of Earth Sciences Vrije Universiteit Amsterdam Amsterdam The Netherlands; ^6^ Laboratory of Geoinformation Science and Remote Sensing Wageningen University and Research Wageningen The Netherlands; ^7^ Met Office Hadley Centre Exeter UK; ^8^ Geography Department Humboldt‐Universität zu Berlin Berlin Germany; ^9^ Integrative Research Institute on Transformations of Human‐Environment Systems (IRI THESys) Humboldt‐Universität zu Berlin Berlin Germany; ^10^ Georges Lemaître Center for Earth and Climate Research, Earth and Life Institute Université Catholique de Louvain & F.R.S.‐FNRS Louvain‐la‐Neuve Belgium; ^11^ F.R.S.‐FNRS Brussels Belgium

**Keywords:** climate, croplands, Earth observations, Earth system models, forestry, grazing, land management, land use

## Abstract

As the applications of Earth system models (ESMs) move from general climate projections toward questions of mitigation and adaptation, the inclusion of land management practices in these models becomes crucial. We carried out a survey among modeling groups to show an evolution from models able only to deal with land‐cover change to more sophisticated approaches that allow also for the partial integration of land management changes. For the longer term a comprehensive land management representation can be anticipated for all major models. To guide the prioritization of implementation, we evaluate ten land management practices—forestry harvest, tree species selection, grazing and mowing harvest, crop harvest, crop species selection, irrigation, wetland drainage, fertilization, tillage, and fire—for (1) their importance on the Earth system, (2) the possibility of implementing them in state‐of‐the‐art ESMs, and (3) availability of required input data. Matching these criteria, we identify “low‐hanging fruits” for the inclusion in ESMs, such as basic implementations of crop and forestry harvest and fertilization. We also identify research requirements for specific communities to address the remaining land management practices. Data availability severely hampers modeling the most extensive land management practice, grazing and mowing harvest, and is a limiting factor for a comprehensive implementation of most other practices. Inadequate process understanding hampers even a basic assessment of crop species selection and tillage effects. The need for multiple advanced model structures will be the challenge for a comprehensive implementation of most practices but considerable synergy can be gained using the same structures for different practices. A continuous and closer collaboration of the modeling, Earth observation, and land system science communities is thus required to achieve the inclusion of land management in ESMs.

## 1 INTRODUCTION

Three quarters of the Earth's ice‐free land surface are in some form managed by humans (Luyssaert et al., 2014). While this provides essential food, fiber, energy, and living space for about 7 billion people (Haberl et al., 2007), the extent and magnitude of land‐use change impacts key Earth system processes, including the climate, in major ways. Global climate change has been accelerated by greenhouse‐gas emissions from land‐use changes, with about one‐third of all anthropogenic CO_2_ emissions over the industrial era attributable to deforestation (Houghton, 2003). In addition, changes in albedo, energy fluxes, and water fluxes induce changes in surface climate as important locally as those induced by the increased global greenhouse‐gas concentration (de Noblet‐Ducoudré et al., 2012), which can feed back on atmospheric dynamics on regional scale (Winckler, Reick, & Pongratz, 2017). Understanding how different types of land‐use change affect climate‐relevant parameters is therefore important.

**Figure 1:**
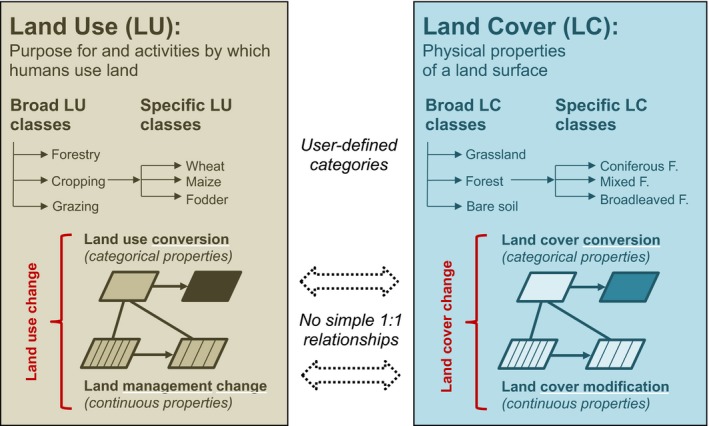
Clarifying land‐use terminology. Clarifying basic terminology in terms of land‐use change and land‐cover change is essential given that these terms are not always used consistently in the climate change and land system science communities. Land cover is defined as the sum of all land surface properties at a given location (e.g., biophysical, morphological, topographical) and typically described by vegetation and soil characteristics at that location. Land cover is often categorized in broad land‐cover classes (e.g., forest, grassland, bare ground), which can be subdivided into more detailed classes (e.g., deciduous forest, coniferous forest, mixed forest). Land‐cover maps can provide one class label or a continuous class proportion (e.g., % tree cover) for each gridcell. Land use relates to the purposes or functions that humans assign to a given location and how humans interact with the land. Land use is also typically categorized in broad classes (e.g., forestry, grazing, cropping). Land management refers to the land‐use practices that take place within these broader land‐use classes (e.g., sowing, fertilizing, weeding, harvesting, thinning, clear‐cutting). Land‐use change over time then refers to either (a) conversions among broad land‐use classes (e.g., agricultural expansion) or (b) changes in land management within these classes (e.g., agricultural intensification). Importantly, both types of land‐use changes can result in either (i) land‐cover conversion from one class to another (e.g., forest loss), or (ii) in more subtle changes in ecosystem properties (e.g., forest degradation), denoted as land‐cover modifications. Some previous studies and reviews (e.g., Erb, Luyssaert, et al., 2016; Luyssaert et al., 2014) used simplified terminology, assuming that land‐use conversion always lead to land‐cover conversion, and land management changes to land‐cover modifications. While this is often the case, it is important to note that the terms land cover and land use are not congruent, as land management can lead to land‐cover conversions (e.g., wood harvesting resulting in the full clear‐cutting of forest), and land‐use conversion can happen without drastic changes in land cover (e.g., putting livestock on natural grasslands)

This requires bridging different Earth system science disciplines, particularly the climate change and land system science communities, and establishing a common terminology. Here, we use the term land‐use change as an umbrella to entail both conversion from one broad land‐use class to another (e.g., from forestry to cropping) and changes in land management within one land‐use class (e.g., intensification of cropping). Importantly, this moves beyond simplified definitions that defined land‐use change by its impact on land cover to define land‐use change more comprehensively and mechanistically (see Figure 1). Both land‐use conversions and land management impact the climate through biogeochemical and biogeophysical pathways.

Earth system models (ESMs) have become key tools to assess how land‐use change has affected the climate in the historical past and how it may affect the climate for future scenarios. The Coupled Model Intercomparison Project 5 (CMIP5), which provided the simulations underlying the Intergovernmental Panel on Climate Change (IPCC) 5th Assessment Report, was the first CMIP that included spatially explicit maps of land‐use change as a forcing (Hurtt et al., 2011) in addition to industrial greenhouse‐gas fluxes. At this stage, models were limited in the types of land‐use change represented: Most models represented anthropogenic conversions in land use, typically those that also result in land‐cover conversions such as the clearing of natural vegetation for cropland expansion (Boysen et al., 2014; Brovkin et al., 2013). These are relevant for about 18‐29% of the ice‐free land surface, while land management, inducing both land‐cover conversions and modifications, affects about 71%–76% of the land (Luyssaert et al., 2014). Nevertheless, the effects of land management were practically absent in CMIP5. Only some ESMs accounted for land management practices like wood harvest (e.g., Shevliakova et al., 2013; Wilkenskjeld, Kloster, Pongratz, Raddatz, & Reick, 2014).

However, observational evidence points toward land management inducing important effects on surface climate (Luyssaert et al., 2014). Individual modeling studies confirm that land management practices such as irrigation (Boucher, Myhre, & Myhre, 2004), crop harvest (e.g., Pugh et al., 2015), no‐till (Davin, Seneviratne, Ciais, Olioso, & Wang, 2014), grazing (Eastman, Coughenour, & Pielke, 2001), or forestry practices (Naudts et al., 2016) can notably alter biogeophysical properties and biogeochemical cycles in large regions of the world. A recent comparison study of several land surface models (LSMs) for certain land management practices revealed that emissions from land use may be consistently underestimated by earlier assessments accounting only for anthropogenic land‐cover conversions (Arneth et al., 2017), challenging our understanding of terrestrial carbon sources and sinks.

Beyond this evidence of important effects on the Earth system land management becomes increasingly important in the context of climate policy. Land use in general is a tool to mitigate global climate change (UNFCCC, 2005). Given that intensification will play a decisive role in fulfilling the surging future demand for land‐based food, feed, and fiber (Erb, Lauk, et al., 2016; Foley et al., 2011), land management choices provide a key lever for future mitigation and adaptation (Erb, Haberl, & Plutzar, 2012; Smith et al., 2013). A key issue is to assess the trade‐offs between intensification through changes in land management and further expansion into natural ecosystems (Kuemmerle et al., 2013; Lambin et al., 2013). Policy decisions around land‐based mitigation activities, such as biofuel, need to be informed by both biogeochemical and biophysical implications of such actions.

For these reasons moving beyond conversions in land use that induce land‐cover conversions to also represent land management has become a key priority for Earth system modeling. This is also reflected in additional data layers provided for CMIP6 and proposed simulations that isolate management effects and compare them across models (Lawrence et al., 2016). Assessments of regional land management strategies with promise to help mitigate and/or adapt to climate change are envisaged within the Land Use Model Intercomparison Project (LUMIP) (Lawrence et al., 2016).

A model extension toward land management further provides a direct link between ESMs and integrated assessment modeling (IAM) by sharing common input and output variables, such as amount of irrigation and fertilization or forest and agricultural yields. Land management thus provides a way to test and improve the consistency between these two types of models. IAMs and ESMs have so far been linked only loosely due to both methodological and data challenges (Prestele et al., 2017) and the fact that feedbacks between environmental and human systems in many cases are small (Van Vuuren et al., 2012). However, land management and land‐use conversions are a prime example for where these feedbacks may be non‐negligible because of the tight coupling of the land surface with the atmosphere and of the land surface state with human decision making (Van Vuuren et al., 2012). First attempts at synchronously coupling IAMs and ESMs therefore exist (e.g., Collins et al., 2015) and show, for example, a decrease in projected managed area when beneficial effects on plant productivity such as increasing atmospheric CO_2_ levels are accounted for (Thornton et al., 2017).

Given that human and computational resources are limited, ESM groups need to prioritize which management practices should be implemented preferentially. This process can be guided by the following criteria:

1. Model or observation‐based evidence shows that the effects of a land management practice on the Earth system are substantial.
2. The spatial extent that a land management practice covers is large.
3. Processes relating a land management practice to its biophysical and biogeochemical effects need to be sufficiently understood to be implementable in a process‐based model.
4. The current concepts and structures underlying ESMs are sufficient or can easily be adapted to capture the land management practice.
5. The data required to drive ESMs extended by a land management practice need to be available. Also, specific evaluation datasets would ideally be available.

The first two of these criteria are related to the prospective impact of the land management practice on the Earth system, the third and fourth to the implementation in ESMs, with the last relating to data availability for any realistic simulation. Studies have assessed the spatial extent of various practices and gathered evidence of land management effects (see Luyssaert et al., 2014; and Erb, Luyssaert, et al., 2016; for reviews). A recent study has reviewed the current state of knowledge of major land management practices with respect to the level of process understanding of Earth system impacts and data availability of the underlying drivers (Erb, Luyssaert, et al., 2016).

We lack, however, an assessment of ways to implement land management practices in current ESM structures. Further, data availability needs to be matched with modeling needs to guide prioritization in the observational community for collection of additional datasets. This study will address these gaps. Here, we focus on implementing the ten land management practices that were selected by Erb, Luyssaert, et al. (2016) based on their global prevalence across a diversity of biomes and the strength of their biogeophysical and biogeochemical effects on the Earth system, as described in the literature. These 10 land management practices are: (1) forestry harvest; (2) tree species selection; (3) grazing and mowing harvest; (4) crop harvest and crop residue management; (5) crop species selection; (6) fertilization of cropland and grazing land; (7) tillage; (8) crop irrigation (including paddy rice irrigation); (9) artificial drainage of wetlands for agricultural purposes; and (10) fire as a management tool. We will discuss the status of implementation of land management in ESMs, possible implementation approaches for these ten practices, and data availability for model input and evaluation. This study will thus identify challenges and opportunities for the assessment of land management effects in Earth system research and allow for a comprehensive prioritization of various land management practices.

## 2 STATUS OF LAND MANAGEMENT IN EARTH SYSTEM MODELS

### 2.1 Current state of implementation

**Figure 2:**
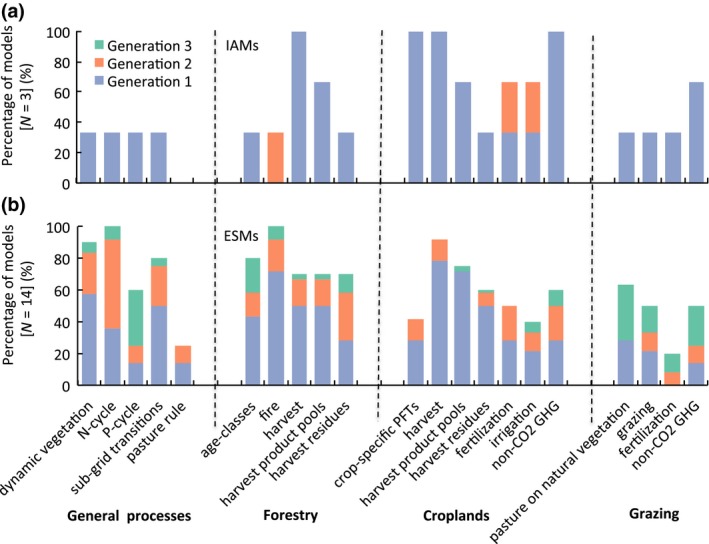
Percentage of (a) integrated assessment models and (b) Earth system models representing various processes related to the conversion in land use or land management. The different colors indicate different generations of models: present use (Generation 1), current development cycle (Generation 2), and plans beyond the Coupled Model Intercomparison Project 6 (CMIP6) (Generation 3)

We conducted a survey among modeling groups participating in international studies including land‐use change (SOM text S1). Hence, by design, all 17 models who participated currently represent land‐use change in some form. Prior to the inclusion of land‐use change, ESMs typically already included a submodel for natural vegetation processes, to account for processes such as changes in biogeographical distribution of natural vegetation or wildfires (Figure 2). Yet, this does not imply that the link between natural processes and land‐use change is well developed. For instance, models disagree on if and how fire should be represented on managed areas (e.g., Rabin et al., 2017) and few models feature an explicit interaction of natural and anthropogenic land‐cover modifications, such as the preferential allocation of pasture on natural grasslands (e.g., Schneck, Reick, Pongratz, & Gayler, 2015).

As climate models moved toward ESMs, the carbon cycle was included to prognostically calculate the atmospheric CO_2_ concentration and capture this dominant driver of anthropogenic climate change (Flato et al., 2013). The carbon cycle is thus represented more frequently than nitrogen or phosphorus cycles. In fact, simulated land surface emissions of non‐CO_2_ greenhouse gases are implemented in only around one‐third of all models considered in our survey (Figure 2). Forest and crop harvest and the corresponding product pools as well as subgrid scale transitions, which all directly alter vegetation and soil carbon stocks, are the most common processes related to land management that are considered in current ESMs (Figure 2). It is worth emphasizing that the development of increasingly complex biophysical models on the one hand and biogeochemistry models on the other hand not necessarily implies that the two are integrated. For example, only three of nine participating models also include tree age classes, but a representation of forest structure is needed to capture the biophysical effects of wood harvest in addition to the biogeochemical effect (e.g., Otto et al., 2014). Similarly, models might represent the release of carbon from fires while the feedback on the biophysical part through albedo may not exist in the model (e.g., Lasslop, Thonicke, & Kloster, 2014).

Despite the small number of IAMs participating in our survey, some clear differences and common features emerge in the treatment of land management between ESMs and IAMs: Forest and crop harvest are important practices also in IAMs (Figure 2), although the focus is on their socioeconomic importance, rather than for carbon cycling as in the ESMs. This different focus of IAMs and ESMs is reflected in the subordinate role of processes related to natural vegetation and vegetation dynamics in IA modeling (Figure 2).

### 2.2 Planned implementations

Ongoing activities aim at moving ESMs toward an extension of biogeochemical cycles and greenhouse‐gas fluxes beyond carbon, but also to implement more detail on agricultural management (Figure 2). Some land management practices, such as fertilization and irrigation, are well captured by crop models (e.g., Brisson et al., 2003), and currently move to the focus of the ESM community due to the emerging empirical evidence of their substantial biogeophysical (in particular for irrigation) and biogeochemical effects (in particular for fertilization, grazing, and residue management) (see Erb, Luyssaert, et al., 2016, for a detailed review).

**Figure 3:**
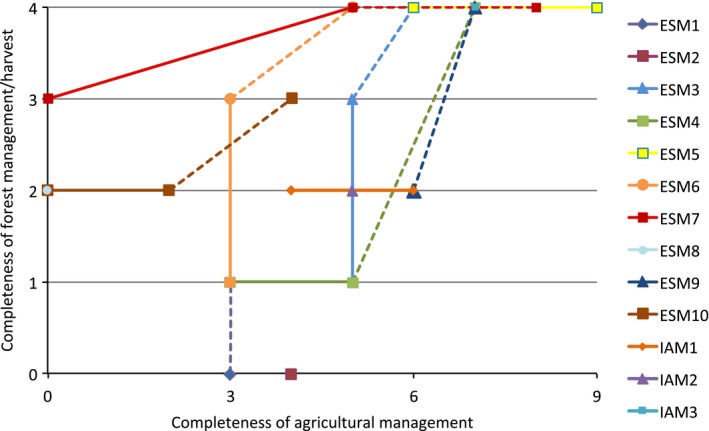
Representation of land management in ten Earth system and three integrated assessment models (all models from our survey (text S1) that provided information for all three development cycles). Solid lines connect present use and current development cycle, dashed lines current development cycle with plans beyond CMIP6. The completeness score reflects how many of forest management and agricultural management processes and variables are considered in the models. For forest management the maximum score of four reflects that (1) wood harvest, (2) forest age classes, (3) the fate of harvest, and (4) the fate of residues are considered. For agricultural management the maximum score of nine would include (1) cropland presentation, (2) crop harvest, (3) fate of harvest, (4) crop residues, (5) fertilizer use, (6) use of irrigation, (7) inclusion of other greenhouse gases, (8) grazing, and (9) pasture management. A score of zero means that none of them are included

**Figure 4:**
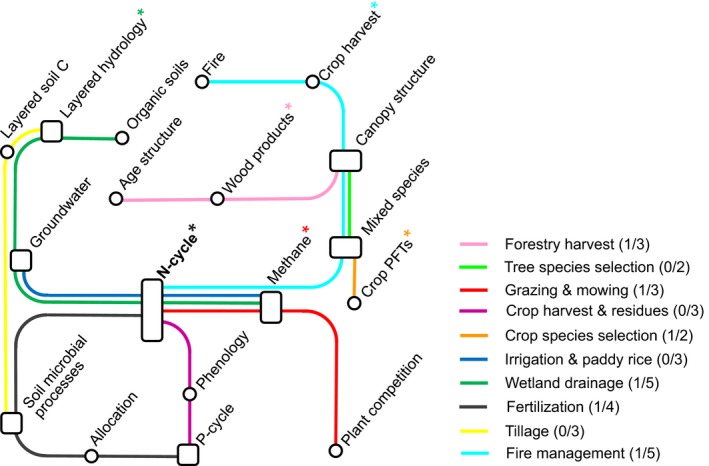
Dependencies of processes and structures required for the comprehensive implementation of the ten discussed land management practices. Asterisks indicate which processes and structures are also needed for a basic implementation. The number of processes and structures required for a basic and comprehensive implementation are indicated for each practice in parentheses. Processes and structures for both basic and comprehensive implementation are collected from the description of implementation of land management practices in Section 3.1

With these plans, there is a clear trend toward a more complete representation of land‐use change, including land management effects, in ESMs from the current state to the perspective beyond CMIP6 (Figure 3). A tendency appears for models to compensate for their “weaknesses” first by improving on the aspect, either forestry or agricultural management, which was more coarsely represented before (Figure 3). It needs to be noted that development paths across models have similarities because dependencies of certain processes on others are the same for all models, for example, the requirement of a nitrogen cycle for fertilization (see also Figure 4). The long‐term consequence of the planned developments is a convergence of models toward a detailed representation of land management. However, on the timescales covered here (several years beyond CMIP6) the diversity and amount of processes that can be represented in the face of limited resources will keep models dissimilar (the models do not converge yet at the highest complexity in Figure 3). Representation of the same process also differs between models, meaning that even models with the same degree of complexity can exhibit marked differences in their simulated behavior and sensitivity. The development path taken by each model to add a more comprehensive representation of land management thus differs based on current capabilities and different prioritization.

While it must be expected that in the medium term, as models start to implement different land management practices, model spread will increase, the common trend toward high complexity (Figure 3) suggests that models eventually converge on a more homogeneous accounting for land‐use conversions and land management processes alike. Currently, studies applying several LSMs for their estimates of land‐use change impacts on surface climate and biogeochemical fluxes typically rely on multimodel mean and spread for a best estimate (e.g., Le Quéré et al., 2015) despite the fact that the amount and types of land‐use and land management practices differ across models. For example, in LUCID‐CMIP5 (“Land‐Use and Climate, IDentification of robust impacts” in CMIP5) only three of six models accounted for pasture as a specific plant functional type, three models accounted for subgrid scale transitions, and one model accounted for wood harvest (Brovkin et al., 2013). The inclusion of more land management practices will in the future allow to form larger ensembles of models that account for the same processes, as currently part of the spread across models must still be attributed to different land‐use change processes included (Houghton et al., 2012). Moving toward a greater comprehensiveness will also facilitate evaluation of models against observations. For instance, Nyawira, Nabel, Don, Brovkin, and Pongratz (2016) found that the observed sign of soil carbon changes for forest‐cropland transitions could be simulated by a LSM only after the inclusion of crop harvesting.

## 3 IMPLEMENTATION OF TEN LAND MANAGEMENT PRACTICES IN EARTH SYSTEM MODELS

### 3.1 Basic and comprehensive implementation approaches

We outline possible approaches of implementation of the ten management practices to assess the required implementation efforts and associated need for data. The starting point for the suggested approaches is a typical land surface component of an ESM that is capable of representing land‐cover conversions (Boysen et al., 2014; Brovkin et al., 2013). We acknowledge that individual models may differ substantially in their process description, but common histories and fundamental approaches (Fisher, Huntzinger, Schwalm, & Sitch, 2014) allow for capturing common features across a wide range of models. While many effects of certain land management practices require no structures beyond those contained in typical LSMs, but only additional detail (e.g., the introduction of product carbon pools for harvested material in parallel to existing soil and vegetation carbon pools), other effects require processes to be implemented that were previously ignored or implicitly parameterized (such as prognostic groundwater storage).

Since the representation of a land management practice in a model can vary substantially in its level of comprehensiveness, we describe possible approaches on two levels: A “basic” implementation aims at capturing some of the most obvious effects of a practice (e.g., biomass removal for forest harvest), i.e., fulfills the minimum requirement for a model to account for this practice. A more “comprehensive” implementation accounts for details of how the practice is realized (e.g., harvesting of differently aged forest and influencing canopy structure). The aim of the latter is a comprehensive depiction of effects on the Earth system, although some of these effects are marginal (Erb, Luyssaert, et al., 2016). As the key purpose of assessing basic and comprehensive implementations is to span the range of methods for the prioritization, the exact distinction is not crucial. Table S2 summarizes the need of additional input data.

#### 3.1.1 Forestry harvest

Wood harvest is a major process controlling carbon stocks and physical structure of managed forests. A basic representation of wood harvest in ESMs could simply remove a prescribed amount of carbon each year (e.g., Shevliakova et al., 2009). This could be implemented as a mass removal of carbon from the biomass pool or by a removal of a fraction of biomass in the forest area according to harvested area. In the simplest schemes there may be no biophysical impact, although in some models carbon stocks are linked to structure via, for example, canopy height or leaf area and so some biophysical effects may be included. The main effect, though, of a simple scheme is the loss of biomass from the forest. This may be released straight to the atmosphere or stored for some period in a product pool or pools. In order to drive the simple scheme, wood harvest amounts are required (as either mass or area harvested). Slightly more complex schemes may also treat the fate of harvest residue as input to litter or soil carbon pools. The scheme could be evaluated using remote‐sensing‐based biomass products or land‐based forest inventories (Roman‐Cuesta et al., 2016).

A more complex scheme for wood harvest may target specific tree age or size for harvesting, in which case age or size classes within the forest must already be represented (Bellassen, Le Maire, Dhôte, Ciais, & Viovy, 2010). Such a scheme would capture the effect of rotation length on carbon storage and may then be able to represent more complete effects on the physical structure of the forest such as on canopy structure or leaf area (Naudts et al., 2015). This would enable biophysical forcing via albedo or roughness changes (Otto et al., 2014; Raupach, 1994). The comprehensive approach could include some more specific forest management practices like thinning, coppice or short rotation coppice. Some data exist on forest management strategy and age or size to cut (see Text S2), and evaluation could use basal area maps (de Rigo, Caudullo, Busetto, & San Miguel, 2014) or tree height products (Lefsky, 2010; Simard, Pinto, Fisher, & Baccini, 2011). MODIS data could be used to evaluate changes in albedo (Moody, King, Platnick, Schaaf, & Gao, 2005).

#### 3.1.2 Tree species selection

The tendency of foresters to select economically interesting species can be captured by adding species‐specific plant functional types (PFTs) to the existing PFTs representing unmanaged forests in ESMs. Besides species‐specific parameter sets, this approach does not require any additional model changes. Tree‐species‐specific parameter sets should include parameters related to carbon allocation, nitrogen cycling, photosynthesis, surface albedo, phenology, and evapotranspiration (Farley, Jobbágy, & Jackson, 2005; Kirschbaum et al., 2011). Depending on the forestry harvest scheme, parameters related to wood harvest, for example, harvest age or size, should also be included. Species‐specific parameters regarding disease, pest and drought resistance (e.g., mortality) could help to capture carbon releases due to forest dieback. If short rotation coppice is a management strategy a specific PFT could be dedicated to the species that are usually used in these plantations. The species‐specific parameterization will affect all processes that are implemented at the PFT level. If the model has a multilayer soil carbon and hydrology scheme (see Section 3.1.7), species‐specific root profiles will help to capture species differences in water and nutrient uptake. For the main European tree species parameter sets have already been derived and applied (Hickler et al., 2012; Naudts et al., 2016).

A comprehensive implementation would include the representation of mixed‐species stands. Representing species interactions in mixed stands involves competition for light, water and nutrients (see Pretzsch, Forrester, & Rötzer, 2015 for a review of model approaches). Belowground competition can be captured when the model includes multilayer soil carbon, nitrogen, phosphorus, and hydrology schemes. Capturing light competition, however, would require the replacement of the current “big leaf” approach of most ESMs by a vertically explicit canopy structure with a multilayer radiation scheme (Haverd et al., 2012; McGrath et al., 2016). The combination of a multilayer radiation and energy scheme enables simulating emissions of biogenic volatile organic compounds (Sindelarova et al., 2014). Both basic and comprehensive implementations require tree species distribution, whereas the comprehensive implementation also requires the distribution of mixed stands. The evaluation approach can be similar to the one for forestry harvest (see Section 3.1.1).

#### 3.1.3 Grazing and mowing harvest

While biophysical effects are found to be relatively weak, strong biogeochemical effects relate to this practice, in particular due to the direct effect of carbon removal (Erb, Luyssaert, et al., 2016). The basic implementation and evaluation of effects on carbon stocks are analogous to removal of cropland biomass for crop harvest (e.g., Bondeau et al., 2007; Lindeskog et al., 2013; Shevliakova et al., 2009), requiring information on grazing intensity in terms of amount of biomass or fraction of net primary production (NPP) removed. Effects are limited to those related to altered carbon stocks. Yet, in reality grazing occurs also on shrubby and woody vegetation (affecting low and high vegetation cover, the latter denoted “browsing”), so that additional information is needed on type of vegetation to be grazed (ecosystem type and the share of low and high vegetation affected) to overcome the current common ESM assumption that all land used for grazing is grassland. Such data are scarcely available, adding to the existing uncertainties (Fetzel et al., 2017). An important additional effect of grazing and mowing harvest is the emission of methane from livestock, which accounts for about 2/3 of total non‐CO_2_ greenhouse‐gas emissions from the livestock sector (Herrero et al., 2013). Methane emissions can be simulated by models of different complexity linking feed intake to fermentation products (Chang et al., 2013; Thornton & Herrero, 2010) combined with estimates of number of livestock (FAOSTAT, 2007), but the lack of information on dietary composition in ESMs suggests to approximate this by external input on spatially varying fractions of methane emissions per unit biomass removal.

A more comprehensive implementation accounts for the return of carbon and nutrients in manure and urine, which is important on grazed lands, with consequences on methane and nitrous oxide emissions (Davidson, 2009; Thornton & Herrero, 2010) and on plant productivity by accelerated nutrient cycling (e.g., McNaughton, Banyikwa, & McNaughton, 1997). While methane emissions from manure are commonly quantified as fraction of enteric methane production (Thornton & Herrero, 2010), the simulation of nutrient effects on soil respiration, plant growth, and nitrogen‐related emissions requires a representation of the nitrogen cycle. Information on which systems are grazed vs. mowed is needed to determine manure input, but does not exist yet. Simulating changes in ecosystem structure due to selective grazing such as woody encroachment in semiarid regions, which affects both biogeochemical and biophysical pathways, requires a complex competition scheme.

#### 3.1.4 Crop harvest and residue management

Reflecting crops’ purpose of providing food, feed, and fiber, the most basic implementation just represents a removal of a fixed fraction of biomass at a fixed date (Lindeskog et al., 2013; Shevliakova et al., 2009) or an interception of a fixed fraction of productivity or litter (Olofsson & Hickler, 2008). Removed carbon can be released to the atmosphere under the assumption that consumption of harvested products occurs within short time periods or be transferred to short‐lived soil/litter pools (Oleson et al., 2013; Reick, Raddatz, Brovkin, & Gayler, 2013), such that product pools are dispensable. Consequences of crop harvest are a reduction in vegetation biomass and consequently soil carbon stocks, associated with emissions of CO_2_ to the atmosphere, and biogeophysical changes that are associated with altered vegetation cover. The only required input is information on the amount of biomass that is removed, in absolute terms or relative to standing biomass, although globally fixed rates of removal or biomass left on site are found in model studies (Malyshev, Shevliakova, Stouffer, & Pacala, 2015; Stocker, Strassmann, & Joos, 2011). Evaluation of such removal can be done via yield data (e.g., Food and Agricultural Organization (FAO), 2007) after translating yield dry mass into carbon stocks, and soil carbon chronosequences or paired‐site studies (e.g., Don, Schumacher, & Freibauer, 2011; Poeplau et al., 2011).

More comprehensive implementations will put emphasis on plausible harvest dates by fixing them to statistical information or crop calendars or, in regions with seasonal climate, by interactively simulating harvest dates in dependence on phenological state or climate conditions (e.g., Bondeau et al., 2007; Oleson et al., 2013). Removal of nutrients is simulated together with carbon, which requires particular attention to the magnitude and fate of residues, which return part of the nutrients to the system (Kumar & Goh,^1999^). The outlined approach requires structural changes to the phenology scheme to account for a harvest date, and nutrient cycles. Residual material goes to the litter pools, which generally exist in models; introduction of product pools would allow for accounting for noninstantaneous emissions, for example, due to storage of bioenergy. Effects are changes to carbon and nutrient cycles as well as a more realistic depiction of phenological consequences. Additional input data are needed on fate of harvest and residues and its return to the field as organic amendments. Additional opportunity for evaluation results from the interactive simulation of harvest dates (Sacks, Deryng, Foley, & Ramankutty, 2010).

#### 3.1.5 Crop species selection

The large variety in crop species can be captured by extending the existing model PFTs with crop functional types representing the most widespread agricultural plant traits (Bondeau et al., 2007). If the model already includes crop PFTs, allowing it to treat crops differently than natural vegetation (crop harvest, irrigation, N fertilization, …), no additional structures are needed (Bondeau et al., 2007; Lokupitiya et al., 2009; de Noblet‐Ducoudré et al., 2004). The targeted species‐specific parameters are similar to the ones for tree species selection (see Section 3.1.2); however, additional parameters related to the development of yield‐bearing organs can be included. This approach should allow for capturing crop differences in yield, soil organic matter accumulation, nitrogen and phosphorus uptake, evapotranspiration, and phenology. The latter is important as it includes crop‐specific sowing dates, which can determine harvest date and seasonal changes in albedo (Sacks & Kucharik, 2011). Similar to crop harvest (see Section 3.1.4), evaluation can be done against yield data, and additionally against MODIS albedo and evapotranspiration (Loarie, Lobell, Asner, Mu, & Field, 2011).

The combination of the above described crop‐specific parameterization and the implementation of mixed stands (see comprehensive implementation in Section 3.1.2.) would allow to simulate intercropping (i.e., concurrently growing multiple (crop) species to maximize resource usage) and agroforestry (i.e., if one of the species is a tree) (Brisson, Bussiere, Ozier‐Lafontaine, Tournebize, & Sinoquet, 2004). Agroforestry in different forms may represent an important form of land management, but data are particularly scarce. A recent analysis reveals that about 43% of cropland areas, measured as 1 km^2^ gridcells, had at least 10% tree cover in 2010 (Zomer et al., 2016). A certain fraction of this tree cover consists of patches of cropland interspersed with wood patches, but agroforestry may play a significant role. Beside parameter sets for crop functional types, information on crop type distribution and rotation schemes is needed.

#### 3.1.6 Irrigation and paddy rice

Alleviating the water stress on the vegetation to enhance productivity brings about unintended biophysical and biogeochemical effects, including changes in transpiration, soil albedo and greenhouse‐gas emissions (in particular methane and nitrous oxide) (Erb, Luyssaert, et al., 2016). Most models distinguish between water availability in the soil and the subsequent water status of the plant (e.g., Clark et al., 2011; Krinner et al., 2005; Lawrence et al., 2011; Naudts et al., 2015; Sitch et al., 2003). Hence, a basic implementation could eliminate or reduce water stress by increasing soil moisture at the expense of violating the mass balance closure (Boucher et al., 2004; Leng et al., 2013; de Vrese, Hagemann, & Claussen, 2016). The magnitude of the stress reduction could be prescribed or simulated based on the evapotranspirative demand of the atmosphere (Boucher et al., 2004; Leng et al., 2013; de Vrese et al., 2016). This approach could simulate the effects of irrigation on plant growth, transpiration, soil albedo (Brisson et al., 2003) and other greenhouse‐gas emissions (Kulshreshtha & Junkins, 2001). The approach requires spatially explicit data on irrigation area and fraction of water need fulfilled, as human use does not always correspond to optimal water volume (Döll, Fritsche, Eicker, & Schmied, 2014), and could be evaluated in its effects against yield statistics (e.g., Food and Agricultural Organization (FAO), 2007), remotely sensed phenology (Ganguly, Friedl, Tan, Zhang, & Verma, 2010), and greenhouse‐gas inventories or inversions (Saunois et al., 2016; Thompson et al., 2014).

If mass balance closure is aimed for, which is required for assessments of water availability, the water used for irrigation should, depending on the location, be taken from the simulated aquifers or surface water stocks such as reservoirs and rivers (Gleeson, Wada, Bierkens, & van Beek, 2012; Postel, Daily, & Ehrlich, 1996). This approach is more data‐demanding as the simulated water stocks will need to be evaluated against ground‐truth data. Spatially explicit data on the land area equipped for irrigation and fraction of water need fulfilled should be complemented by data on soil depth to simulate the groundwater table as well as data on sources of extraction. In addition to the evaluation data discussed for the basic approach, this scheme could also be evaluated against statistics of river flow (e.g., Monk, Wood, Hannah, & Wilson, 2007), soil water content (Entekhabi et al., 2010; Tapley, Bettadpur, Ries, Thompson, & Watkins, 2004), and amount of water extracted for irrigation (Gleeson et al., 2012; Postel et al., 1996). Note that we propose to use the amount of extracted water for evaluation rather than for driving the model. This proposition is justified by the fact that changes in aquifers are calculated from irrigation statistics. If irrigation statistics are prescribed, the simulated changes in aquifers can no longer be used to evaluate this aspect of model performance. A consequence of this proposition is that irrigation demand will need to be calculated by considering plant physiology in combination with the atmospheric condition.

A comprehensive implementation of irrigation would also account for paddy rice. Paddy rice has different drivers and effects from irrigation of other crops. The aim is not alleviation of water stress but weed and pest control. The primary impact is via methane emissions. Surface biophysics are also altered in terms of evaporative ability and albedo. To capture emissions of other greenhouse gases, which are particularly important for paddy rice (Wassmann et al., 2000), models should include methane production as well as an N cycle which can adapt to anaerobic conditions through reduced decomposition and enhanced denitrification (Kraus et al., 2015).

#### 3.1.7 Artificial wetland drainage

Wetlands cover about 4% of the land surface but store about one‐third of the soil organic carbon, mainly in peatlands (Aselmann & Crutzen, 1989). Even in the absence of a representation of peatlands, a basic implementation of drainage is feasible in models including a multilayer hydrology by removing water from the lower soil layers and adding it to the run‐off (in addition to the gravitational drainage commonly represented in ESMs). We are not aware of ESMs that implemented wetland drainage.

A comprehensive approach would require that models distinguish between mineral and organic soils (Letts, Roulet, Comer, Skarupa, & Verseghy, 2000/2010; Wisser, Marchenko, Talbot, Treat, & Frolking, 2011) and simulate a groundwater table in its multilayer soil water scheme. Drainage could still be represented by removing water from the soil layer that corresponds to the typical depth of a drainage channel. The effects of a reduced soil water content on greenhouse‐gas emissions and transpiration can then be simulated by the existing process representation in the model. Both basic and comprehensive implementations require knowledge of the extent of drainage and drainage depth and an adequate routing scheme to simulate the lateral transport. Most often drainage is followed by a land‐cover conversion, which is expected to cause the main biogeochemical and biophysical effects. This land‐cover conversion will need to be prescribed unless its socioeconomic drivers are sufficiently understood. The validation of drainage would thus require to separate between the effects of drainage and the subsequent land‐cover change. We could not identify datasets which at present would support such a validation.

#### 3.1.8 Nitrogen and phosphorous fertilization of cropland and grazing land

The biogeochemical effect of N fertilization is large and well‐documented (Erb, Luyssaert, et al., 2016), explaining our suggestion to implement the key N cycling processes in both basic and complex approaches. N cycle processes not related to land management, i.e., representation of N inputs from atmospheric deposition and biological fixation, and ecosystem losses through leaching and microbial emissions, need to be extended to account for input from fertilizer application. In a basic representation, N uptake can be a function of demand and availability, where the demand is determined by assuming a fixed C/N ratio in plant and soil compartments, i.e., if the amount of carbon in the pool increases, N increases accordingly (Goll et al., 2012; Thornton, Lamarque, Rosenbloom, & Mahowald, 2007). In this approach, N limitation is relaxed and biomass production increased as a result of N fertilization, but plants are not allowed to optimize their C/N ratio (Haxeltine & Prentice, 1996), possibly leading to an overestimation of N limitation. In the basic approach microbial N emissions consist of nitrification and denitrification which can be a function of mineral nitrogen concentration, soil moisture, temperature, pH, and carbon availability (Butterbach‐Bahl, Baggs, Dannenmann, Kiese, & Zechmeister‐Boltenstern, 2013).

In the comprehensive implementation N concentration in plant and soil are simulated dynamically, and therefore carbon fluxes respond to the N status (Xu‐Ri & Prentice, 2008; Zaehle & Friend, 2010). In this approach, N uptake can be determined according to Michaelis–Menten kinetics, proportional to fine root biomass or surface area, N availability, and plant N status. Additional model developments could include N‐dependent allocation (shift toward belowground carbon to improve N status, Smith et al., 2014), plant–rhizosphere interactions mediated by carbon export (Stocker et al., 2016), and nitrification and denitrification based on microbial dynamics (Butterbach‐Bahl et al., 2013). The comprehensive implementation should also capture potential biophysical effects of N fertilization through increased leaf area.

Although the effect of fertilization by other nutrients (in particular phosphorus (P) and potassium (K)) on climate is much smaller than for N fertilizer, they could be included if the model includes their respective biogeochemical cycle (e.g., Goll et al. (2012) for phosphorus). Phosphorus in particular plays a key role in the tropics.

The required input for both basic and comprehensive implementations consists of spatially explicit information on the area and amount of applied N (and P, K)‐fertilizer. Additional information could be the timing of the nutrient fertilization. The scheme can be evaluated against yield data (e.g., Food and Agricultural Organization (FAO), 2007), soil carbon (e.g., Don et al., 2011; Poeplau et al., 2011) and N concentration in rivers (Nevison, Hess, Riddick, & Ward, 2016).

#### 3.1.9 Tillage

The multitude of soil processes affected by tillage, which are not well understood (Erb, Luyssaert, et al., 2016), suggest a simple parameterization as basic form of implementation in models (Chatskikh, Hansen, Olesen, & Petersen, 2009). Observation‐based rate modifier terms for reduced tillage and no‐till management for soil respiratory fluxes allow capturing effects on soil carbon stocks and CO_2_ fluxes (Pugh et al., 2015). The only required input is knowledge of the area under tillage and possibly the form of tillage for specific parameterizations. Soil carbon chronosequences or paired sites can be used for evaluation (e.g., Don et al., 2011; Poeplau et al., 2011) if they have not entered the parameterization.

A process‐based representation of tillage effects requires representing vertical layers of soil, with the top layers exchanging carbon and water due to tillage, which leads to different conditions for decomposition. Therefore, the representation of tillage would benefit from a microbial‐based decomposition instead of the first‐order decomposition that is generally used in ESMs but lacks microbial control (Todd‐Brown, Hopkins, Kivlin, Talbot, & Allison, 2012; Xenakis & Williams, 2014). While many LSMs represent vertical soil layers for water (e.g., De Rosnay, Polcher, Bruen, & Laval, 2002; Hagemann & Stacke, 2015; Oleson et al., 2013), layered soil carbon schemes (e.g., Braakhekke et al., 2011; Koven et al., 2013) are less commonly applied. The top‐soil mixing allows for representing altered soil respiration fluxes and, depending on the capabilities of the model's soil scheme, other greenhouse‐gas emissions. Altered soil moisture further influences plant growth, surface water fluxes, and soil albedo. Effects of stubble on albedo can partly be captured by distinguishing between transfer of on‐site residues to soil/litter pools in the case of tillage (see description of crop harvest and residue management) and onsite residues left in the biomass pools for no‐till, or be parameterized, as can be effects of mulch on evaporation (Davin et al., 2014). This more comprehensive implementation, however, requires additional input concerning depth of tillage and possibly seasonal timing. Observational data on soil moisture and albedo for till vs. no‐till locations can be used for evaluation (e.g., Davin et al., 2014) in addition to carbon stock chronosequences or paired sites.

#### 3.1.10 Fire as management tool

Fire has multiple uses in agriculture, for example, to burn crop residues or manage grazing lands. In inhabited fire‐prone areas, prescribed burning is used to prevent wildfires and if these preventive measures fail, fire suppression is expected to avoid losses. In a basic biogeochemistry‐oriented implementation, a fraction of the litter and standing biomass—after harvest—should be put back into the atmosphere as burn gases (Van der Werf et al., 2010). The burn gases could distinguish between different carbon and nitrogen compounds by making use of generic emission factors (Akagi et al., 2011).

A comprehensive implementation accounting for both the biophysical and the biogeochemical effects of fire management (including potentially emissions relevant to wider atmospheric composition interactions such as biomass burning aerosols, methane and carbon monoxide) requires that the vegetation structure is accounted for (Randerson et al., 2006). Where fire management is applied in woodland savannas, this may require structural model developments that enable mixed PFTs (see implementation of mixed stands in Section 3.1.2), such that trees and grasses compete for the same water, nutrient, and light resources (Saito et al., 2014; Scheiter & Higgins, 2009; Simioni, Le Roux, Gignoux, & Sinoquet, 2000). In forests, the vegetation structure needs to represent a canopy structure. Preventive fires will then remove part of the litter and the fuel ladders that connect the litter layer with the top canopy through the crowns of the small trees (Scherer, DAmato, Kern, Palik, & Russell, 2016). The chemical composition and dimensions of the biomass as well as the fire characteristics can be used to adjust the emission factors of the burn gases in terms of their carbon and nitrogen compounds (Lobert et al., 1990; Surawski, Sullivan, Meyer, Roxburgh, & Polglase, 2015; Urbanski, 2013). Both the basic and comprehensive approaches require a spatially explicit driver that prescribes the areas where fire is used as a management tool in agriculture and forestry.

The implementation of fire suppression builds on the functionality required to simulate wildfires (Arora & Boer, 2005; Mann et al., 2016; Thonicke et al., 2010). When a wildfire is ignited and the resources for fire suppression are available, a fire suppression module should stop the fire before the natural conditions for burning would stop the fire. A fire suppression module could thus be driven by regional data on the capacity to fight fires as well as the size of the fire, the population density and the property value in the vicinity of the fire to set decisions rules on where to fight wildfires.

### 3.2 Structural dependencies

The implementation of land management practices might require that new structures and processes are added to the model architecture. In Figure 4 we collect the new structures and processes required for the implementation described in Section 3.1 (in parentheses the total number for basic/comprehensive implementation approaches). The degree of these required changes differs largely between land management practices. For example, tree species selection requires a small, and wetland drainage and fire management a large number of new processes and structures to be implemented.

The required changes introduce dependencies between the land management practices when they are implemented in a comprehensive way, as many processes and structures form basis for more than one practice. Figure 4 shows the interrelation between processes and structures required for the implementation of our ten land management practices. Some structures emerge as being essential in that they form basis for many practices, such as the nitrogen cycle for a more comprehensive implementation, while others are specific to individual practices, such as age structure (Figure 4). Note that the most essential structures of a comprehensive implementation include the nitrogen cycle, canopy structure, mixed‐species stands, and representations of methane, most of which go far beyond an extension of existing structures. Considerations of prioritization thus may not just include the number and complexity of processes and structures required by individual land management practices, but also synergies that practices provide in sharing key structures with other practices. For example, all new structures required for tree species selection would already be available following an implementation of fire management or forestry harvest plus crop species selection; similarly irrigation and paddy rice could use the structures provided by implementation of wetland drainage.

## 4 MATCHING MODEL REQUIREMENTS WITH AVAILABLE DATA

### 4.1 Data requirements and availability

Each land management practice comes with certain additional variables that need to be prescribed from external data (summarized from Section 3.1 in Table S2). Most information is required globally and in a spatially explicit way and describes in particular the extent and intensity of the practices, but implementing crop and tree species selection would also require extending the existing parameter sets. Land management has substantially changed over history, and modeling land‐use change effects involves simulations covering a decadal to centennial timescale to capture in particular delayed biogeochemical fluxes and slow feedback responses in the Earth system. Therefore, input datasets need to cover these timescales. Yet, observational data are useful for model evaluation even when available only for certain regions or time periods.

Timescales of decades to centuries imply that deriving information on the input variables from Earth observation is often not sufficient—it needs to be possible to reconstruct the same variable from statistical or inventory data to capture time periods prior to the satellite era (e.g., statistics on agricultural area by the Food and Agricultural Organization, statistics on wood production by FAO's Global Forest Resources Assessments, forest inventories). Furthermore, ESMs are frequently used to project anthropogenic effects, including land‐use change, into the future (e.g., Brovkin et al., 2013); the same variable thus needs to be available, from global land system or integrated assessment modeling, for future scenarios. For some land‐use conversions and certain land management practices a harmonization of historical, Earth observation, and IAM data has been performed for CMIP5 (Hurtt et al., 2011) and is currently extended for CMIP6, but covers only a subset of the variables identified as essential input in Section 3.1. Discrepancies between priorities of IAMs and ESMs may therefore limit ESM applications for future management.

We assess the availability of observational datasets as input variables or parameters as required by the implementation outlined in Section 3.1. It should be noted that few of these datasets are direct observations—many are processed data products relying on additional assumptions, including partly even process‐based modeling (see Text S2). Our assessment of data availability aims at simulations for the historical time period (e.g., for simulations covering the industrial era) and includes aspects of data quality and spatial and temporal coverage (Table S2 and Text S2). Our assessment reveals that data availability differs vastly across the ten land management practices. Good data availability exists for cropland management practices where mostly area information is required (as for basic implementations of crop species selection, fertilization, tillage). Poor data availability is found for forestry harvest, grazing and mowing harvest, artificial wetland drainage, and tillage when the comprehensive assessment requires additional data streams. Partly this may be attributable to complications in separating natural from managed processes in Earth observations.

### 4.2 Model‐data gaps and opportunities

**Figure 5:**
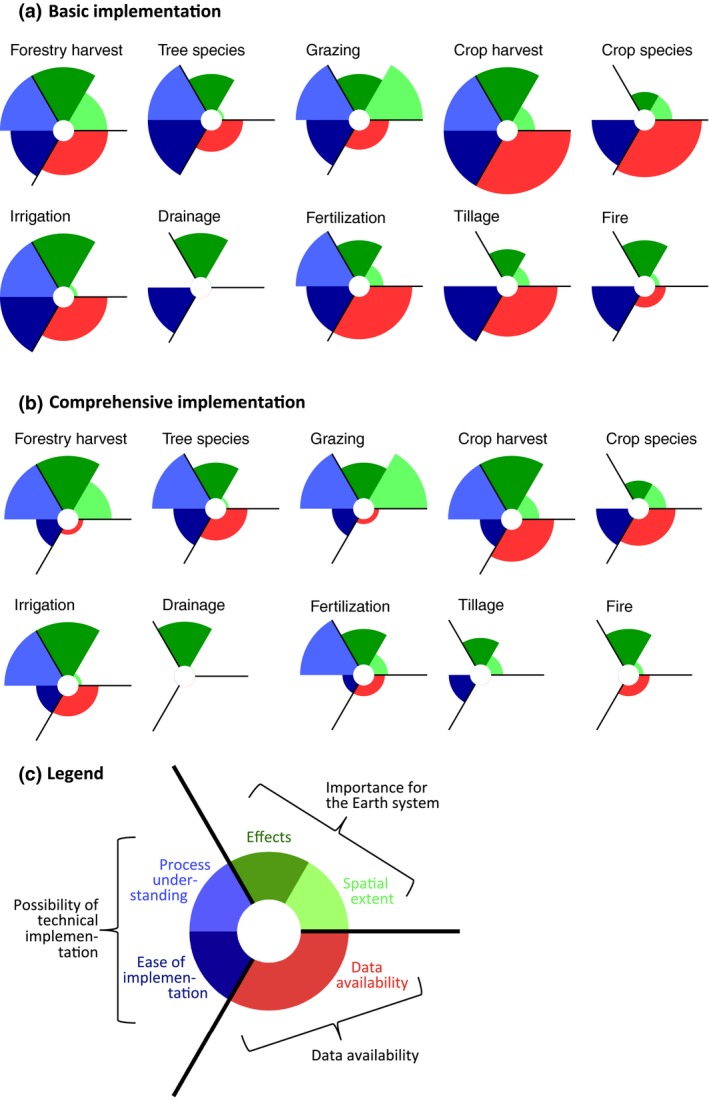
Matching importance for the Earth system, possibility of technical implementation in ESMs, and data availability for the ten land management practices. The legend in panel c explains the criteria: Importance for the Earth system is depicted by spatial extent and combined strength of biogeophysical and biogeochemical effects (from Erb, Luyssaert, et al., 2016 Figures 2 and 3, resp.); possibility of technical implementation in ESMs is depicted by process understanding (classified as either poor or advanced by Erb, Luyssaert, et al., 2016; table 2) and ease of implementation (represented by number of structures required for the implementation approaches of Section 3.1, see also Figure 4); data availability is based on the description and scoring of the individual datasets required for implementation in ESMs as described in Table S2 and Text S2. Panel a refers to a “basic” implementation, b to a “comprehensive” one, meaning that ease of implementation and data availability differ between a and b. All values are scaled to maximum = 1. For data availability, the scorings for the individual variables/datasets are aggregated to one single value as (*N* * 3–SUM(*S*_*i*_))/(*N* * 3–1), where *N* is the number of required datasets (maximum of basic and comprehensive implementation for each land management practice) and S_i_ is the score of the data availability for each dataset i required for the basic and comprehensive implementation, respectively, with 1 = good, 2 = medium, 3 = poor data availability

The criteria for prioritization discussed in the introduction refer to three broad categories: (1) the importance of a land management practice for the Earth system as indicated by spatial extent and strength of biogeochemical and biogeophysical effects, (2) the possibility of technical implementation as indicated by the process understanding and ease of implementation, and (3) data availability. The following conclusions can be drawn by contrasting these three aspects (Figure 5):

1. “Low‐hanging fruits” for modeling a land management practice, where all three aspects are well covered, emerge for a basic implementation approach. Crop harvest and residue management, nitrogen fertilization, and (with some more restrictions on data availability but larger spatial extent) forestry harvest are all important for the Earth system, possible to implement in current ESMs, and provide good data availability (Figure 5a). However, for all three practices the ease of implementation and data availability dramatically drop for a comprehensive implementation (Figure 5b).
2. For some land management practices data availability and robustness is the key obstacle for simulating their effects in ESMs. Most notably, data availability is poor for grazing and mowing harvest, which is important for the Earth system and rather easy to implement in current ESMs. Here, a substantial number of variables needs to be provided with external data and quality of the individual datasets is poor (Figure 5a and Text S2). The problem arises both for the basic and comprehensive implementation, interfering with modeling groups’ plans of moving toward representation of grazing processes in their models (Figure 2). Data availability is poor also for artificial wetland drainage and fire as management tool, but implementation for these practices is equally hindered by the lack of process understanding. Data availability becomes the limiting factor for many land management practices for a comprehensive implementation (Figure 5b).
3. A call for more research on process understanding was voiced by Erb, Luyssaert, et al. (2016) for crop species selection, tillage, artificial wetland drainage, and fire as management tool. Our analysis shows that for the first two, process understanding is indeed the major obstacle, as the available structures of current ESMs are capable of catching the basic effects of crop species selection and tillage and data availability is good. Simulation of the latter two is also hindered by data availability.
4. Existing model structures of current ESMs are largely sufficient to capture key effects of land management, but major extension of current model structures are required to capture both biogeophysical and biogeochemical effects comprehensively.

Our prioritization results are partly reflected in model development: Coincidence of importance, modeling ease, and data availability for a basic implementation of crop harvest and residue management, which only relies on removal of biomass, indeed is one of the most common land management features in the current generation of models (Figure 2). That most models move toward the inclusion of the nitrogen cycle (Figure 2) coincides with nitrogen fertilization being recognized as important and, despite its own complexity, requiring only nutrient‐related structures (Figure 4). Grazing and mowing harvest, which requires many additional structures and processes, is left only to the third generation of ESMs, requiring future progress on data availability. A good observational basis may also have been a driver of past model development. For example, the availability of large databases such as from the eddy covariance network Fluxnet (Baldocchi et al., 2001) or the global plant trait database (Kattge et al., 2011) pushed the carbon cycle development in LSMs; the wide inclusion of wood harvest (Figure 2) occurred as part of the development for CMIP5, which provided gridded wood harvest information (Hurtt et al., 2011). On the other hand, new scientific questions in the ESM community fostered specific development of datasets, such as the first global reconstructions of some historical land‐use and land‐cover conversions (Kaplan, Krumhardt, & Zimmermann, 2009; Klein Goldewijk, 2001; Pongratz, Reick, Raddatz, & Claussen, 2008; Ramankutty & Foley, 1999).

## 5 CONCLUSIONS

As the applications of Earth system models move from general climate projections toward questions of mitigation and adaptation (Lawrence et al., 2016) the more comprehensive representation of land management practices becomes crucial. A corresponding trend toward a more comprehensive representation of land use generally, and land management in particular, in ESMs is clearly discernable. This development can be guided by a prioritization of land management practices based on their importance for the Earth system, the possibility of technical implementation in the model, and data availability. Our review of these aspects reveals some “low‐hanging fruits” such as a basic implementation of crop harvest and residue management, nitrogen fertilization, and forestry harvest, where existing model structures are mostly sufficient to capture certain key effects on the Earth system and the required additional input variables can be derived from observational or statistical datasets.

Our review also pinpoints the need for additional research in specific communities: the implementation, even in a simple form, of the most extensive land management practice—grazing and mowing harvest—is severely hampered by the lack of high‐quality data, and data availability reveals substantial gaps for almost all of the land management practices assessed in this study when the aim is to capture their effects in a comprehensive way. A lack of process understanding is complicating implementation of practices that otherwise can be easily linked to existing model structures and datasets (such as crop species selection and tillage for a basic implementation). Finally, while some key effects of most land management practices can be captured without major extension of ESMs to additional structures, major model development will be the challenge for a comprehensive representation of biogeophysical and biogeochemical effects.

Extending ESMs to a more comprehensive representation of land management effects will in the near future lead to model divergence as the planned paths of model development and prioritization differ between modeling groups. It will, however, allow for a more accurate description of the human impact on the Earth system as long as the multimodel assessments required to overcome model biases are based on selecting models with comparable representation of land management practices. A multitude of observational datasets can be included for evaluation purposes that were not meaningful in earlier‐generation models, because earlier models lacked a detailed representation of vegetation processes that are relevant for land management practices and captured by Earth observations. To achieve a comprehensive inclusion of land management in ESMs a continuous collaboration of the modeling community, Earth observation community, as well as land system science is required beyond the identification of challenges and opportunities provided by this study.

## ACKNOWLEDGEMENTS

We thank the various modeling groups providing insight into their modeling plans as depicted in Figures 2 and 3. The authors gratefully acknowledge the support of the International Space Science Institute (Bern) that sponsored the team on “Integrating Earth Observation data and the description of land management practices into global carbon cycle models” (A.J. Dolman). JP and KN were supported by the German Research Foundation's Emmy Noether Program (PO 1751/1‐1). PM is Research Associate with the Fonds de la Recherche Scientifique (F.R.S.‐FNRS/Belgium), which supports this work. CDJ was supported by the Joint UK BEIS/Defra Met Office Hadley Centre Climate Programme (GA01101) and EU H2020 project CRESCENDO (grant no. 641816) and FP7 LUC4C (grant no. 603542). AJD recognizes the support of the NESSC Netherlands Earth System Sensitivity Centre. MH was supported by the European Space Agency Land Cover CCI Project and the ESA GOFC‐GOLD project office. KHE acknowledges funding from ERC‐Stg 263522 LUISE and H2020 640176 BACI. This work contributes to the Global Land Programme glp.earth. Primary data used in the analysis are archived by the Max Planck Institute for Meteorology and can be obtained by contacting publications@mpimet.mpg.de.
