# Supplementary material for: Models meet data: Challenges and opportunities in implementing land management in Earth system models
Source: Glob Chang Biol. 2017 Dec 13;24(4):1470–87. doi: 10.1111/gcb.13988 (PMC6446815; doi:10.1111/gcb.13988)
Supplement: Supplementary file 3 [file GCB-24-1470-s003.pdf]

## Supplemental Material

### *Text S1: Survey on model status and plans concerning land use and land management*

We conducted a survey among Earth system model (ESM) and land surface model (LSM) groups participating in international studies including land use and land management: ESMs evaluated by Todd-Brown et al. (2013) concerning soil and vegetation carbon cycle representing the model subset of the Coupled Model Intercomparison Project 5 (CMIP5) that includes LSMs with a carbon cycle, models participating in LUCID-CMIP5 ("Land-Use and Climate, IDentification of robust impacts" in CMIP5; Brovkin et al., 2013), and the TRENDY land use simulations of the Global Carbon Project (Le Quéré et al., 2015). Additionally, the four Integrated Assessment Models (IAMs) contributing the Representative Concentration Pathways to CMIP5, which include scenarios of future land-use change (van Vuuren et al., 2011), were invited. Overall, 17 modeling groups responded, of which 11 represent ESMs, 3 uncoupled LSMs, and 3 IAMs.

Questions referred to general model processes relating to vegetation dynamics not specific to land use and land management (e.g., inclusion of nutrient cycles, representation of forest age classes), to land-cover conversions (e.g., if sub-grid transitions are considered), and to land management on forest, crop, and pasture (e.g., if wood/crop harvest is considered), see Tab. S1. All questions were posed for three different time periods: (1) A present state that refers to use and usage up to now, e.g. in projects like CMIP5, TRENDY, or ISI-MIP; (2) the current development cycle, including CMIP6; (3) future ambitions beyond CMIP6. Only 15 and 13, respectively, of the 17 modeling groups reported plans for the current development cycle and beyond CMIP6.

### References

Brovkin, V., Boysen, L., Arora, V., Boisier, J., Cadule, P., Chini, L., . . . others (2013). Effect of anthropogenic land-use and land-cover changes on climate and land carbon storage in CMIP5 projections for the twenty-first century. Journal of Climate, 26(18), 6859–6881.

Le Quéré, C., Moriarty, R., Andrew, R. M., Canadell, J. G., Sitch, S., Korsbakken, J. I., . . . others (2015). Global carbon budget 2015. Earth System Science Data, 7(2), 349–396.

Todd-Brown, K., Randerson, J., Post, W., Hoffman, F., Tarnocai, C., Schuur, E., & Allison, S. (2013). Causes of variation in soil carbon simulations from CMIP5 Earth system models and comparison with observations. Biogeosciences, 10(3).

Van Vuuren, D. P., Edmonds, J., Kainuma, M., Riahi, K., Thomson, A., Hibbard, K., . . . others (2011). The representative concentration pathways: an overview. Climatic Change, 109(1–2), 5.
