# Supplementary material for: Models meet data: Challenges and opportunities in implementing land management in Earth system models
Source: Glob Chang Biol. 2017 Dec 13;24(4):1470–87. doi: 10.1111/gcb.13988 (PMC6446815; doi:10.1111/gcb.13988)
Supplement: Supplementary file 4 [file GCB-24-1470-s004.docx]

***Text S2: Supplemental information to Tab. S2. This text provides information of the datasets available for each variable that was identified to be required for implementing a management practice into an Earth system model.***

**General remarks on observational data for modelling and judgement of data availability**

It should be noted that few of the datasets discussed below are pure observational data. Nevertheless, some remote sensing products that provide variables directly related to the radiative fluxes measured by the sensor are indeed direct observations, such as albedo (Moody et al., 2005) or fluorescence (Frankenberg et al., 2011). Similarly, compilations of local vegetation properties like the trait database (Kattge et al., 2011) mostly provide direct observational data. Most other datasets, however, are processed data products which are informed by data but go beyond directly observable quantities in that they rely substantially on additional assumptions. Examples are leaf area derived from remote sensing, where external datasets on land cover type are used to transform the spectral information to leaf area index estimates (Myneni et al., 2002) or products of turbulent heat fluxes derived by upscaling Fluxnet observations to global coverage using machine learning algorithms that link the heat fluxes with climate, meteorological and land cover information (Jung et al., 2011). Extensive use of additional assumptions based on statistical or even process-based relationships will result in model-based data products, such as MODIS-based estimates of net primary production (NPP). It should therefore be kept in mind that direct observations, processed data products and model-based data products are not distinct categories but can all be used to drive, parameterize, and/or validate Earth System Models (ESMs) by allowing to reconstruct the required variable at the required temporal, spatial or categorical scale.

The majority of observations and data products share common features that are shortcomings in the context of usage with ESMs. As outlined in the previous paragraph, reliance on additional assumptions – often to fill spatial and temporal gaps in the available data – introduce additional uncertainties. Furthermore, field measurements are often biased towards the Northern hemisphere, in particular North America and Europe (e.g., Baldocchi et al., 2001). Datasets covering more than a few decades usually rely on more assumptions in distant compared to recent decades. For example, reconstructions of agricultural areas can use almost three decades of satellite products, six decades of statistical data (from the Food and Agricultural Organization, FAO), but have to rely on semi-empirical relationships with population (Klein Goldewijk et al., 2016) prior to 1960. Country statistics such as those collected by the FAO suffer from changing national boundaries, with statistical data self-reported by the countries differing in quality and definition. When describing the individual data below, we did not repeat these general issues but did take them into account when assessing their uncertainty. Uncertainties of observations and data products, however, usually cannot be quantified in a robust and comparable way. Therefore, the score for data availability indicated in parenthesis is based on expert judgment of the information described with each dataset.

Our expert judgement is inevitably subjective to some extent, but scoring followed a systematic approach and for each data stream the following was taken into account: if

- the spatial coverage is local (scored 1), continental (scored 2) or global (scored 3) (local observations or reconstructions may be appropriate for parameterization, if all relevant environmental zones are sufficiently covered, but input for model drivers requires maps);
- the temporal coverage is a (usually present-day) snapshot (scored 1), is of decadal scale with some possibility to reconstruct further back in time (scored 2), or is of the centennial scale required in particular for simulating the biogeochemical effects of land management practices (scored 3);
- the resolution is on national level only, requiring proxy information on the spatial pattern that is less or more available (scored 1 or 2) or spatially explicit and high (relative to the typical resolution of ESMs of several degrees) (scored 3);
- the variable provided by the datasets is only a proxy related to the variable of interest (e.g., area equipped for irrigation instead of actually irrigated area), which may be more or less hard to transform to the required variable (scored 1 or 2) or the variable itself (scored 3);
- the quality of the dataset is judged low (scored 1), moderate (scored 2) or high (scored 3) based on the data source and level of processing as outlined before.

This information is summarized in table S2.

1. **Forestry harvest**

**a. Wood harvest amount by area or mass (medium)**

*Purpose of the reconstruction in ESMs (see Sec. 3.1.1 in the main text):* If forest management has been internalized in the ESM and the ESM thus simulates wood harvest as a function of forest growth and/or forest structure (for example Naudts et al., 2015), a wood harvest reconstruction could be used to validate the internal forestry module. If such a module is absent, a wood harvest reconstruction could drive the model by prescribing the wood that needs to be harvested (for example Shevliakova et al., 2009).

*Available data for the reconstruction:* Harvest reconstructions can rely on six decades of global statistics documenting the harvested wood volume as compiled, archived and shared by the FAO (http://www.fao.org/forestry/statistics/en/) and FAO Forest Resource Assessment (FRA) (http://www.fao.org/forest-resources-assessment/en/). For the last three decades national forest inventory data are widely available, mainly for developed countries with a substantial wood-based economy (Vilén et al., 2012), and have been used to refine harvest reconstructions. Harvest concessions are available for some countries from Global Forest Watch (http://www.globalforestwatch.org/). Remote sensing data provide a measure of the forest area with an abrupt decrease in canopy cover (Mildrexler et al., 2007) which in regions with little natural disturbance could be attributed to forest management.

*Available reconstructions:* (1) Wood harvest expressed as mass and area has been reconstructed globally with a 0.5 degree resolution for the years 850 to 2005 (Hurtt et al., 2011). The reconstruction integrates the FAO data (back to 1961) and model simulations (Hurtt et al., 2011). Since its first release, the reconstruction has been updated for the Coupled Model Intercomparison Project 6 (Lawrence et al., 2016; Hurtt et al., 2017). The updated version goes until 2015 at 0.25 degree resolution. (2) Wood harvest expressed as mass and area has been reconstructed globally at 1 km for the year 2000 (Roman-Cuesta et al., 2016). The reconstruction integrates National Forest Inventory, FAO FRA and a remote-sensing-based global land cover assessment (GLC, 2000).

*Specific limitations:* Currently available data and measurements likely overlook the use of wood as fuel wood, which is often obtained from tree-bearing ecosystems outside forests (Krausmann et al., 2008; Bais et al., 2015). These reconstructions typically consider that all wood is obtained from harvesting forests, while in reality significant amounts of wood, especially in historical periods and pre-industrial economies, can be obtained from other land covers (farmlands, pastures, orchards, roadsides…). The available reconstructions do not account for differences in harvest intensity between age classes and tree species. The dynamics of wood harvest in the Hurtt et al. datasets in terms of area are derived from wood harvest statistics that were in turn derived by dividing harvest volumes with potential biomass density, modeled on basis of an extension of the empirical MIAMI model (Lieth, 1975; Hurtt et al., 2002), which will not be consistent with other vegetation models that the dataset is applied with.

**b. Age-class information for wood harvest (poor)**

*Purpose of the reconstruction in ESMs (see Sec. 3.1.1 in the main text):* If forest management has been internalized in the ESM and the ESM thus simulates wood harvest as a function of forest growth and/or forest structure (for example Naudts et al., 2015), an age-class reconstruction could be used to validate the internal forestry module. If such a module is absent, a wood age-class reconstruction could drive the model by prescribing the age class and PFTs that need to be harvested (for example Shevliakova et al., 2009). For both approaches towards forest management an age-class reconstruction could be used as the boundary condition for a spin-up.

*Available data for the reconstruction:* Measures of forest age are common in national forest inventories but rarely go back more than three decades (Vilén et al., 2012). Stand-replacing disturbances leave a fingerprint on the age structure. Such forest inventory data can be complemented by national disturbance databases, e.g., the Canadian Large-Fire database, or by remote sensing-based disturbance indices (Mildrexler et al., 2007). Backcasting can be applied to reconstruct the age class structure 50 to 100 years prior to the earliest observations.

*Available reconstructions:* A global age reconstruction of forest age for the year 2010 at 0.5 degree spatial resolution is underway (Poulter et al., in prep.). Regional reconstructions already exist for Europe, Canada, and the USA. For 25 European countries the age structure of the forest has been reconstructed for every decade between 1950 and 2010 for a 0.25 degree spatial resolution (Vilén et al., 2012). For the year 2003, a 1 km x 1 km reconstruction was compiled for North America excluding Alaska and Mexico (Pan et al., 2011).

*Specific limitations:* In the absence of stand-replacing disturbances such as fires, storms or clear cuts – as in the cores of the humid tropical forests – forest age is ill-defined or of little relevance.

**c. Fate of harvest and residues (poor)**

*Purpose of the reconstruction in ESMs (see Sec. 3.1.1 in the main text):* Reconstruction of the fate of the harvest and residues could be used to parameterize residue management and wood use.

*Available data for the reconstruction:* Reconstructions of the fate of harvest can rely on six decades of global statistics documenting the wood use as compiled, archived and shared by the FAO (http://www.fao.org/forestry/statistics/en/). We are not aware of large-scale data compilations that are readily available to reconstruct the volume and fate of harvest residues left on-site. The volume and/or mass of the residues left on-site could be estimated by combining national forest inventory data (standing volume) and harvest statistics (removed volume). The difference would be an estimate for the residues left on-site.

*Available reconstructions:* The available reconstruction of the amount and area of wood harvest (see section 1a) distinguishes 3 to 5 coarse classes of wood use such as industrial roundwood and fuelwood (Hurtt et al. 2017, Roman-Cuesta et al. 2016). For details on the spatial and temporal resolution of these reconstructions see section 1a. We are not aware of reconstructions of the amount and fate of the residues left on-site.

*Specific limitations:* N/A

**d. Forest management maps (poor)**

*Purpose of the reconstruction in ESMs (see Sec. 3.1.1 in the main text):* If forest management has been internalized in the ESM and the ESM thus simulates wood harvest as a function of forest growth and/or forest structure (for example Naudts et al 2015), a management reconstruction is required to drive the management module of the ESM. If forest management is driven by a harvest reconstruction (for example Brovkin et al., 2013), there is no use for a forest management reconstruction.

*Available data for the reconstruction:* Direct measures of forest management are limited to a few national forest inventories and rarely go back more than three decades. Past management, however, has left a fingerprint on the present day forest structure. Such current age structures can be used to inform 100 to 150 years of management reconstructions. Longer forest management reconstructions can be informed by combining population reconstructions and anecdotic historical evidence (see McGrath et al., 2015 for examples)*.* Biotic (e.g., site index), abiotic (e.g., accessibility), socio-economic (e.g., proximity to a city) and political (e.g., protected land) site information can constrain past, present and future management options.

*Available reconstructions:* (1) The authors are not aware of a global forest management reconstruction (2) European forest management reconstruction from 1600 to 2010 at 0.5 degrees (McGrath et al., 2015). Between 1600 and 1830 the reconstruction relies on reconstructed wood demand. Following 1830 the reconstruction makes use of the current age class structure which in turn is based on forest inventory data. (3) European assessment of the most rational forest management strategy around the year 2000 based on biological, socio-economic and political characteristics of a location (Hengeveld et al., 2012).

*Specific limitations:* Currently available data likely overlook the contribution of tree-bearing ecosystems outside forests (Krausmann et al., 2008; Bais et al., 2015), trade as well as the contribution of substitution of wood products (such as manure burning, solar energy, recycling).

1. **Tree species selection**

**a. Parameter sets for species-PFTs (good)**

*Purpose of the reconstruction in ESMs (see Sec. 3.1.2 in the main text):* When tree species rather than plant functional types are to be modelled, the model needs to be parameterized at the species level to account for species-specific ecophysiological process rates as well as differences in vulnerability and management.

*Available data for the reconstruction:* The TRY database on traits (Kattge et al., 2011) currently contains almost three million trait entries for 69,000 out of the world's 300,000 plant species, with a focus on 52 groups of traits characterizing the vegetative and regeneration stages of the plant life cycle, including growth, dispersal, establishment and persistence. Not all species-specific parameters needed in land surface models are related to plant traits, hence, several thousands of research papers contain parameter values that are not contained in the TRY database.

*Available reconstructions:* The database contains contemporary traits only and covers all biomes. Temporal reconstructions are not available.

*Specific limitations:* N/A

**b. Maps of dominant species (areas) (poor)**

*Purpose of the reconstruction in ESMs (see Sec. 3.1.2 in the main text):* Species maps are an essential driver when simulations at the species level are aimed for for regions where species distribution is driven by humans rather than edaphic controls.

*Available data for the reconstruction:* Measures of the dominant tree species are included in all national forest inventories but those rarely go back more than three decades (Vilén et al., 2012). Records from pollen (Trondman et al., 2015; Fyfe et al., 2015), charcoal (Robin et al., 2014) and genetic records (Petit 2002, 2008) contain comprehensive evidence to reconstruct species distributions for the past 10 ka.

*Available reconstructions:* There are no global reconstructions of tree species distribution and we are not aware of ongoing efforts. Regional datasets exists for 17 European countries (Brus et al., 2011) around the year 2000 at a 1 km x 1 km resolution, for Russia around the year 2000 at a 1 km x 1 km resolution (Bartalev et al., 2004; Yaroshenko et al., 2005); for China in the 1990s at a 10 km by 10 km resolution (Guo, 2010), and the USA between 2001 and 2003 at a 0.25 km x 0.25 km resolution (Blackard et al 2008).

*Specific limitations:* Despite hyper-dominance in, for example Amazonian rainforest (ter Steege et al., 2013), the concept of dominant tree species is of little practical use in tropical forests with a large species diversity.

1. **Grazing and mowing harvest**
   1. **Type of vegetation grazed or mown (medium)**

*Purpose of the reconstruction in ESMs (see Sec. 3.1.3 in the main text):* Information on grazing extent or amount of grazed biomass alone is not sufficient information for ESMs to represent grazing and mowing harvest because grazing can occur on different types of vegetation, including shrubby and woody vegetation. A reconstruction of type of vegetation grazed or mown is thus needed to overcome the current common ESM assumption that all land used for grazing is grassland.

*Available data for the reconstruction:* National data on permanent pastures is available from FAO statistics (1961 onwards), gridded information on the extent of grazing land is also available (Haberl et al., 2007; Ramankutty et al., 2008; Fetzel et al., 2017), albeit characterized by large discrepancies between datasets. All datasets have a global coverage and use the year 2000 reference year. Information on ecosystems subject to grazing can be derived from overlay of gridded grazing land maps on existing maps of potential natural vegetation (e.g., Ramankutty and Foley, 1999) in regions where a certain biome dominates, but requires additional information for mixed biomes. Time series of ecosystem changes are not available.

*Available reconstructions:* HYDE 3.2 (Klein Goldewijk et al., 2016) provide information on the extent of rangeland and managed pastures, with the first indicating that the vegetation type dynamically simulated by the ESM remains in the first case, but is replaced by managed grassland in the second case. The allocation of country-level pasture and grazing land follows probability rules based on region and type of remote-sensing derived land cover classes. Another set of rules based on land cover class determines whether or not natural vegetation is transformed to grassland. The HYDE 3.2 reconstruction has global coverage with 5 arc min. spatial resolution and covers multiple centuries and millennia.

*Specific limitations:* Data uncertainty is very large, owing to uncertainties in the input data (derived from national FAO data; Ramankutty et al., 2008; Fetzel et al., 2017), spatial downscaling approaches and (semantic as well as spatial) discrepancies between biome maps (Ramankutty and Foley, 1999; Olson et al., 2001; Simons et al., 2001). Information on grazed/moved fractions of plants (e.g. woody vs. herbaceous plant parts) is not available, but would be required for the representation of ecological processes.

- 1. **Biomass removal in grazing or mowing in mass or fraction of NPP (poor)**

*Purpose of the reconstruction in ESMs (see Sec. 3.1.3 in the main text):* Global data and reconstructions of biomass harvest due to grazing and mowing or of biomass removal as a fraction of NPP is needed in ESMs to determine the amount of carbon to be removed by this land management practice.

*Available data for the reconstruction:* No global data are available on the amount of biomass grazed by livestock or ruminants (including wild animals). Only few national statistics do report grazed biomass volumes (e.g. hay production), thus most available data are reconstructed based on feed demand models.

*Available reconstructions:* Gridded biomass removal are available for the year 2000 only (Haberl et al., 2007; Herrrero et al., 2013; Fetzel et al., 2017; for drylands: Petz et al., 2014) at the resolution of 5 arc minutes; time series are missing. Theses data are based on feed demand modelling and the so-called “grazing-gap” method (Krausmann et al., 2008; Bouwman et al., 2005).

*Specific limitations:* Uncertainties on the extent of grazing land as well as on harvested volumes are massive (Fetzel et al, under review) and warrant caution when using global datasets.

- 1. **CH4 per unit removed biomass (poor)**

*Purpose of the reconstruction in ESMs (see Sec. 3.1.3 in the main text*): The lack of information on type and dietary composition of ruminants in ESMs suggests to approximate methane emissions by external input on spatially varying fractions of methane emissions per unit biomass removal instead of applying more complex modelling linking feed intake to fermentation products.

*Available data for the reconstruction:* No direct observational or statistical data of this variable is available.

*Available reconstructions:* Reconstructions on the amount of CH4 released per unit of biomass are available for the year 2000 (Herrero et al., 2013), based on livestock density maps (Wint and Robinson, 2007), a feed balance model and a metabolism model based on stoichiometry. These global maps have a spatial resolution of 1 km by 1 km. Simple reconstructions use Tier-1 per-head values and species specific emission rates (IPCC) and information on livestock density. Currently, no time series is available. The information could be constructed from information on harvest volumes and the type of forage which could be approximated by browsed vegetation units. An intricacy relates to the use of cropland-derived feedstuff that can distort the picture.

*Specific limitations:* N/A

- 1. **Extent of grazing vs mowing (poor)**

*Purpose of the reconstruction in ESMs (see Sec. 3.1.3 in the main text):* The distinction of grazing vs. mowing would allow to improve the representation of ecological flows, e.g. of litter and on-site manuring, and thus improve the link between aboveground and below-ground processes.

*Available data for the reconstruction:* No data available.

*Available reconstructions:* No global reconstruction is currently available due to absence of empirical data.

*Specific limitations:* N/A

1. **Crop harvest and residue management**
   1. **Biomass removal in mass or fraction of NPP (good)**

*Purpose of the reconstruction in ESMs (see Sec. 3.1.4 in the main text):* Global data and reconstructions of biomass harvest due to crop harvest or of biomass removal as a fraction of NPP is needed in ESMs to determine the amount of carbon to be removed by this land management practice. If harvested amount is prescribed not in absolute numbers but relative to productivity or standing biomass, information on yields can be used for evaluation.

*Available data for the reconstruction:* Yearly national crop harvest data are available at FAOSTAT (http://www.fao.org/faostat/en/) for the period 1961 to today. Yearly subnational and sub-subnational data can be used from various national and international statistic agencies. A list of statistical agencies that provide subnational and sub-subnational crop harvest data can be found in the supplement information of Ray et al. (2012) and in Monfreda et al. (2008).

*Available reconstructions:* Hurtt et al. (2017) provide a global reconstruction at 0.25 degree spatial resolution on harvest and yields for the period 850 AD to 2015 AD; Ray et al. (2012) provide crop harvest and yields as global spatially explicit reconstruction at 5 arc min. spatial resolution from 1961 to 2008. Iizumi and Ramankutty (2016) provide global crop harvest and yields for the period 1981 to 2010 at 1.125 degree spatial resolution. Regional data products can be found in Brisson et al. (2010) and Lin and Huybers (2012). Brisson et al. (2010) provide yearly national and subnational crop harvest and yield information from 1950 to 2010 for eight European countries, Lin and Huybers (2012) yearly national crop harvest and yield information from 1950 to 2010 for 47 different countries. For two of these countries, France and the United States, the information is provided on sub-national scale.

*Specific limitations:* All reconstructions provide harvest and yield information that can be converted into biomass removal in mass or fraction of NPP. Hurtt et al. (2017) relies entirely on FAO statistics for its reconstruction, but covers the total crop harvest and yield of all crop species. A distinction between the species is not made. Ray et al. (2012) and Iizumi and Ramankutty (2016) used extensive subnational crop harvest data and crop species for further spatial disaggregation. However, these reconstructions cover only major crop species, namely wheat, soybean, rice and maize. Together, these four crop species cover 57% of world-wide consumed calories (Tilman et al., 2011). The regional reconstructions of Brisson et al. (2010) and Lin and Huybers (2012) provide the total crop harvest and yield per country, but make no distinction between certain crop types. Both data products are in a non-gridded format. No reconstruction or international data set provides inter-annual crop harvest information.

- 1. **Fate of harvest and residue (medium)**

*Purpose of the reconstruction in ESMs (see Sec. 3.1.4 in the main text):* Reconstruction of the fate of the harvest and residues could be used to parameterize residue management, which is particularly important for estimating removal of nutrients in addition to that of carbon or their return to the field as organic amendments. Information on longer-term storage, e.g. for bioenergy, can be accounted for by diverting the harvested material into product pools of different lifetimes in ESMs.

*Available data for the reconstruction:* No direct observational or statistical data of this variable is available beyond local scale.

*Available reconstructions:* For the year 2000, information on the flow of cropland biomass from production to consumption is available at the level of countries (Krausmann et al., 2008; Smith et al., 2013). These data contain information on the use of cropland residues (e.g. straw left on field or harvested for litter in the livestock sector or bioenergy), derived from top-down considerations and few case studies (Wirsenius 2003; Krausmann et al., 2008). Time series for the 20^th^ century (period 1910 to 2005) are also available on the national level only, derived from census statistics and expert guess (Krausmann et al., 2013).

*Specific limitations:* N/A

1. **Crop species selection**
   1. **Parameter sets for species-PFTs (good)**

*Purpose of the reconstruction in ESMs (see Sec. 3.1.5 in the main text):* When crop species rather than plant functional types are to be modelled, the model needs to be parameterized at the species level to account for species-specific ecophysiological process rates as well as differences in vulnerability and management.

*Available data for the reconstruction:* The TRY database on traits (Kattge et al., 2011) -data repository currently contains almost three million trait entries for 69,000 out of the world's 300,000 plant species, with a focus on 52 groups of traits characterizing the vegetative and regeneration stages of the plant life cycle, including growth, dispersal, establishment and persistence. Not all species-specific parameters needed in land surface models are related to plant traits, hence, several thousands of research papers contain parameter values that are not contained in the TRY database.

*Available reconstructions:* the database contains contemporary traits only. Temporal reconstructions are not available.

*Specific limitations:* N/A

- 1. **Maps of species (areas) (good)**

*Purpose of the reconstruction in ESMs (see Sec. 3.1.5 in the main text):* Maps of crop species allow for region-specific accounting of crop functional types based on the fractional share of a grid-cell.

*Available data for the reconstruction:* Yearly national crop data are available at FAOSTAT (http://www.fao.org/faostat/en/) for the period 1961 to today. Yearly subnational and sub-subnational data can be used from various national and international statistic agencies. A list of statistical agencies that provide subnational and sub-subnational crop data can be found in the supplement information of Ray et al. (2012). These data bases distinguish all crop species types.

*Available reconstructions:* For the year 2000, Monfreda et al. (2008) provide information for 175 cultivars at a resolution of 5 arc min. The study by Ray et al. (2012) provide a reconstruction of four main crop species (wheat, soybean, maize and rice) for the period 1961 to 2008 at 5 arc min. spatial resolution. Iizumi and Ramankutty (2016) reconstructed the same crop species types for the period 1981 to 2010 at 1.125 degree spatial resolution. HYDE 3.2 (Klein Goldewijk et al., 2016) modelled a reconstruction of global rice distribution for longer time spans (back to 10,000 BC) on 5 arc minute spatial resolution. All reconstructions have global coverage.

*Specific limitations:* No reconstruction distinguishes all crop species types. A more comprehensive coverage of crop species is only available for the year 2000 (Monfreda et al., 2008).

- 1. **Maps of mixed cropping and rotation schemes (poor)**

*Purpose of the reconstruction in ESMs (see Sec. 3.1.5 in the main text):* In combination with crop-specific parameterization, maps of mixed cropping systems would allow models that are capable of representing mixed stands to represent intercropping and agroforestry.

*Available data for the reconstruction:* No globally available information is currently available.

*Available reconstructions:* No global reconstruction is currently available due to absence of empirical data.

*Specific limitations:* N/A

1. **Irrigation (including paddy rice)**
   1. **Irrigated area (good)**

*Purpose of the reconstruction in ESMs (see Sec. 3.1.6 in the main text):* Independent of the modelling approach representing irrigation as direct alleviation of a water stress function or by addition of water the ESMs needs information on where this irrigation is applied.

*Available data for the reconstruction:* FAO records are available of area equipped for irrigation from 1961 to present at FAOSTAT (<http://www.fao.org/faostat/en/>).

*Available reconstructions:* Siebert et al. (2015) provides estimates for the period from 1900 to 2005. Before 1960 these estimates are based on historical archives. Global maps provided by Siebert et al. (2015) have a spatial resolution of 5 arc minutes. MODIS-based maps available from Salmon et al. (2015) will be implemented in the MODIS collection 6 product and are to be released soon. These global maps are available for the year 2005 at 500 m spatial resolution. Hurtt et al. (2017) includes a dataset of units fraction of crop area irrigated, differentiating the irrigated fraction of C3, C4, annual and perennial crop area. These estimates are given in 0.25 degree spatial resolution and are based on the HYDE 3.2 data base (Klein Goldewijk et al., 2016). HYDE 3.2. bases its estimates on the above-listed data sources. For the period 1700 to 1900 estimates were taken from Siebert (2008), extrapolated based on per-capita relationships. Spatial resolution of HYDE 3.2 for irrigation is 5 arc minutes. Irrigation datasets exist for recent and past periods (Freydank and Siebert, 2008; Siebert et al., 2015) and are relatively robust in terms of the total area at country level.

*Specific limitations:* Uncertainties related to spatial patterns for the historical reconstructions are large (Salmon et al., 2015). Furthermore, all data relate to areas equipped for irrigation, and not the actual water used, which can lead to distortion when assessing Earth system effects (see below).

- 1. **Fraction of water need fulfilled (poor)**

*Purpose of the reconstruction in ESMs (see Sec. 3.1.6 in the main text):* As water usage depends on economic factors, water requirements cannot be endogenized in ESMs and external information is needed on how much the plants’ water stress should be alleviated or how much water is added by irrigation.

*Available data for the reconstruction:* FAO's Aquastat database (http://www.fao.org/nr/water/aquastat/main/indexfra.stm) has data on water withdrawal for irrigation starting in 1958, but these data are very incomplete in terms of country and year coverages. Furthermore, they do not directly measure the amount of water actually delivered to crops, as this variable also includes losses along the irrigation system. This variable may thus serve to validate the amount of water needed for irrigation for some countries and years, but cannot serve as a driver.

*Available reconstructions:* There is currently no reconstruction of actual water use for irrigation which is informed by historical data. Available products such as the assessments done for water footprinting (Aldaya et al., 2012) rely on models, such as the CROPWAT model from FAO, which estimates water needs for irrigation based on maps of areas equipped for irrigation (see above) and either a simple relaxation of water constraints on plant growth or different options of irrigation schedules.

*Specific limitations:* No dataset thus currently exists that would indicate the fraction of the agricultural water need fulfilled by irrigation at large scales.

- 1. **Area of paddy rice (medium)**

*Purpose of the reconstruction in ESMs (see Sec. 3.1.6 in the main text):* Because paddy rice has very different effects from irrigation of other crops (such as methane emissions), its extent needs to be separately known to drive the ESMs.

*Available data for the reconstruction:* Yearly national crop data are available at FAOSTAT (http://www.fao.org/faostat/en/) for the period 1961 to today. Yearly subnational and sub-subnational data can be used from various national and international statistic agencies. A list of statistical agencies that provide subnational and sub-subnational crop data can be found in the supplement information of Ray et al. (2012). These data bases distinguish all crop species types and thus provide the area of paddy rice as harvested area.

*Available reconstructions:* The reconstruction by Ray et al. (2012) provides a reconstruction of paddy rice for the period 1961 to 2008 at 5 arc min. spatial resolution. Iizumi and Ramankutty (2016) reconstructed paddy rice for the period 1981 to 2010 at 1.125 degree spatial resolution. HYDE 3.2 (Klein Goldewijk et al., 2016) modelled a reconstruction of global paddy rice distribution for longer time spans (back to 10,000 BC) on 5 arc minute spatial resolution. All reconstructions have global coverage.

*Specific limitations:* Only Klein Goldewijk et al. (2016) covers a longer time span than the FAO period. For the period before FAO data was available (before 1960) some rice growing countries were taken from (Mitchell, 2007a, b, c) for the 1890-1950 CE period. The rest was hind-casted on a per capita basis for countries were no historical data could be found (Klein Goldewijk et al., 2016).

1. **Artificial wetland drainage**
   1. **Drained area (poor)**

*Purpose of the reconstruction in ESMs (see Sec. 3.1.7 in the main text):* Independent of the exact modelling approach drainage is represented as removing water from a soil layer, which requires information on the regions where this happens.

*Available data for the reconstruction:* Datasets on drainage area at country level are available from FAO (FAO AQUASTAT, 1994, 2003, 2004), ICID (2005) and CEMAGREF (2005), which are based on various data sources.

*Available reconstructions:* Feick et al. (2005) provided a global map for the year 1990 of artificially drained agricultural areas, identifying the fraction of each 5 min by 5 min cell area that is equipped for improved drainage.

*Specific limitations:* The main limitation is that the dataset is only available for one time period around 1990. Further, as for irrigation, the dataset indicates “area equipped”, which does not necessarily mean these areas as indeed actively managed for drainage, which induces large uncertainties in the area that is actually managed.

- 1. **Depth of drainage (poor)**

*Purpose of the reconstruction in ESMs (see Sec. 3.1.7 in the main text):* Independent of the exact modelling approach drainage is represented as removing water from a or several soil layer(s), which requires information on the depth of drainage.

*Available data for the reconstruction:* No data available.

*Available reconstructions:* No global reconstruction is currently available due to absence of empirical data.

*Specific limitations:* N/A

1. **Nitrogen fertilization**
   1. **Area fertilized (medium)**

*Purpose of the reconstruction in ESMs* *(see Sec. 3.1.8 in the main text):* Different from e.g. biological N fixation artificial fertilizer application cannot be endogenized in ESMs due to its dependence on economic factors. Both area and amount of fertilizer application are needed to inform the models’ nutrient module about additional nutrient availability.

*Available data for the reconstruction:* Yearly national-scale fertilizer data (area and amount) are globally available from FAOSTAT (http://www.fao.org/faostat/en/) for the period 1961 to today. The database distinguishes different fertilizer types (e.g., artificial and manure). Crop-specific, national-scale fertilizer application rates for two major fertilizers (N and P) for 88 different countries can be found in the International Fertilizer Industry Association (IFA) (data set name: ‘‘Fertilizer Use by Crop 2002,’’ available by request from FAO). The IFA data cover a period from 1994 to 2001. Together with a crop distribution map this information can be used to disentangle fertilization area and rate (amount) per crop type. Several data bases with subnational fertilizer information (area and amount) exist, for example USDA (2013) and Eurostat (http://ec.europa.eu/eurostat/web/agri-environmental-indicators/data/database).

*Available reconstructions:* Hurtt et al. (2017) provides a global reconstruction at 0.25 degree spatial resolution providing fraction of crop area fertilized, differentiating the fertilized fraction of C3, C4, annual and perennial crop area for the period 850 AD to 2015 AD. Potter et al. (2010) reconstructed global fertilizer application, differentiating between different fertilizer types for the year 2000 at 5 arc minutes spatial resolution.

*Specific limitations:* The reconstruction by Hurtt et al. (2017) is based on FAO only for recent decades. Extrapolation back in time to centennial timescales comes with large uncertainties as intensity of fertilization and fertilizing regimes (e.g., green manuring vs mineral fertilizer) has been subject to substantial changes over time. Also Potter et al. (2010) based their reconstruction largely on FAO data. Available subnational data, such as from USDA or Eurostat was yet not used in any reconstruction, but would provide more spatial detail and more data for the reconstruction.

- 1. **Amount of fertilizer (medium)**

*Purpose of the reconstruction in ESMs (see Sec. 3.1.8 in the main text):* Different from e.g. biological N fixation artificial fertilizer application cannot be endogenized in ESMs due to its dependence on economic factors. Both area and amount of fertilizer application are needed to inform the models’ nutrient module about additional nutrient availability.

*Available data for the reconstruction:* Same as for area fertilized.

*Available reconstructions:* Same as for area fertilized.

*Specific limitations:* Same as for area fertilized.

- 1. **Timing of fertilization (poor)**

*Purpose of the reconstruction in ESMs (see Sec. 3.1.8 in the main text):* As nutrient uptake and allocation depend on the state of the vegetation, timing of fertilizer application may be useful as additional input for a more detailed representation of fertilization.

*Available data for the reconstruction:* No data available.

*Available reconstructions:* No global reconstruction is currently available due to lack of empirical data.

*Specific limitations:* N/A

1. **Tillage**
   1. **Area tilled (poor)**

*Purpose of the reconstruction in ESMs (see Sec. 3.1.9 in the main text):* Independent of the modelling approach representing tillage via a simple rate modifier term for soil respiration or in a process-based way the ESMs needs information on where tillage occurs.

*Available data for the reconstruction:* No data available.

*Available reconstructions:* No global reconstruction is currently available due to absence of empirical data.

*Specific limitations:* N/A

- 1. **Depth of tillage (poor)**

*Purpose of the reconstruction in ESMs (see Sec. 3.1.9 in the main text):* A process-based representation of tillage in ESMs will build on a multi-layer water and carbon scheme. Data on depth of tillage informs the model which layers are directly disturbed.

*Available data for the reconstruction:* No data available.

*Available reconstructions:* No global reconstruction is currently available due to absence of empirical data.

*Specific limitations:* N/A

- 1. **Timing of tilling (poor)**

*Purpose of the reconstruction in ESMs (see Sec. 3.1.9 in the main text):* A detailed implementation of tillage in ESMs captures effects on microbial decomposition, soil moisture and albedo, all of which exhibit seasonal changes. Therefore knowledge on the timing of tilling will improve ESM simulations.

*Available data for the reconstruction:* No data available.

*Available reconstructions:* No global reconstruction is currently available due to absence of empirical data.

*Specific limitations:* N/A

1. **Fire as management tool**
   1. **Agricultural area burned (medium)**

*Purpose of the reconstruction in ESMs (see Sec. 3.1.10 in the main text):* Because fire depends on other than just natural factors when applied as management tool on agricultural areas ESMs need external information on the extent to which it is applied*.*

*Available data for the reconstruction:* Satellite data is the primary source of data on global land use and fire and falls into three primary categories: detection of active fires, mapping of post-fire burned areas (fire scars), and fire characterization (e.g. fire severity, energy released). For the purposes of the reconstruction in ESMs burned area products are relevant. The most commonly used are those from MODIS, the MODIS global active fire product (MCD14) and burned areas product (MCD45, MCD64) (Giglio et al., 2013), but there are other ones as well (e.g., Alonso-Canas and Chuvieco, 2015). These yearly products are available at 0.25 degree spatial resolution from 1995 until present.

*Available reconstructions:* These maps are produced annually and can be overlaid with ancillary (static) land cover maps to derive the agricultural area burned.

*Specific limitations:* However, separation between human-induced and naturally induced fires (e.g. ignition in savannas used for grazing) is not possible on basis of these datasets, but assessments for recent points in time at larger spatial scales exist (e.g. Lauk and Erb, 2009). Longer (decadal-centennial) time series are not available.

- 1. **Fraction of biomass to return to atmosphere upon burning (poor)**

*Purpose of the reconstruction in ESMs (see Sec. 3.1.10 in the main text):* In a basic implementation of fire management a certain fraction of the biomass on the burned area, prescribed from external data, would be returned to the atmosphere*.*

*Available data for the reconstruction:* No data available.

*Available reconstructions:* Such information can in theory be derived from using the global fire products and additional information. One example is the Global Fire Emissions Database (GFED) that combines satellite information on fire activity and modelled vegetation productivity to estimate gridded monthly burned area and fire emissions, as well as scalars that can be used to calculate higher temporal resolution emissions (van der Werf et al., 2010). However, little has been done to derive the fraction of biomass to return to atmosphere upon burning as a direct input variable and data availability in that regard remains poor.

*Specific limitations:* N/A

- 1. **Information on suppression of natural fires (poor)**

*Purpose of the reconstruction in ESMs (see Sec. 3.1.10 in the main text):* Data on suppression of natural fires could be used as input in ESMs to act on the simulation of wildfires.

*Available data for the reconstruction:* No data available.

*Available reconstructions:* No global reconstruction is currently available due to absence of empirical data.

*Specific limitations:* N/A

**References**

Aldaya, M. M., Chapagain, A. K., Hoekstra, A. Y., & Mekonnen, M. M. (2012). The water footprint assessment manual: Setting the global standard. Routledge.

Alonso-Canas, I., & Chuvieco, E. (2015). Global burned area mapping from ENVISAT-MERIS and MODIS active fire data. Remote Sensing of Environment, 163, 140–152.

Bais, A. L. S., Lauk, C., Kastner, T., & Erb, K. (2015). Global patterns and trends of wood harvest and use between 1990 and 2010. Ecological Economics, 119, 326–337.

Baldocchi, D., Falge, E., Gu, L., Olson, R., Hollinger, D., Running, S., ... others (2001). FLUXNET: A new tool to study the temporal and spatial variability of ecosystem–scale carbon dioxide, water vapor, and energy flux densities. Bulletin of the American Meteorological Society, 82(11), 2415–2434. Bartalev, S., Ershov, D., Isaev, A., Potapov, P., Turubanova, S., & A.Yu., Y.

(2004). Russia’s forests: dominating forest types and their canopy density; scale 1:14,000,000. Space Research Institute of the Russian Academy of Sciences (RAN), Forest Ecology and Production Center of the Russian Academy of Sciences (RAN), Global Forest Watch, Greenpeace Russia, Moscow.

Blackard, J., Finco, M., Helmer, E., Holden, G., Hoppus, M., Jacobs, D., . . . others (2008). Mapping US forest biomass using nationwide forest inventory data and moderate resolution information. Remote Sensing of Environment, 112(4), 1658–1677.

Bouwman, A., Van der Hoek, K., Eickhout, B., & Soenario, I. (2005). Exploring changes in world ruminant production systems. Agricultural Systems, 84(2), 121–153.

Brisson, N., Gate, P., Gouache, D., Charmet, G., Oury, F.-X., & Huard, F. (2010). Why are wheat yields stagnating in Europe? A comprehensive data analysis for France. Field Crops Research, 119(1), 201–212.

Brovkin, V., Boysen, L., Arora, V., Boisier, J., Cadule, P., Chini, L., . . . others (2013). Effect of anthropogenic land-use and land-cover changes on climate and land carbon storage in CMIP5 projections for the twenty-first century. Journal of Climate, 26(18), 6859–6881.

Brus, D., Hengeveld, G., Walvoort, D., Goedhart, P., Heidema, A., Nabuurs, G., & Gunia, K. (2011). Statistical mapping of tree species over Europe. European Journal of Forest Research, 131(1), 145–157.

CEMAGREF. (2005). International drainage database. Dataset available at: http://drainage.montpellier.cemagref.fr/drainage systems.php.

FAO AQUASTAT 1994–2004. (2005). Datasets available at: http://www.fao.org/ag/agl/aglw/aquastat/dbase/index.stm.

Feick, S., Siebert, S., & Döll, P. (2005). A digital global map of artificially drained agricultural areas. Frankfurt Hydrology Paper.

Fetzel, T., Havlik, P., Herrero, M., & Erb, K.-H. (2017). Seasonality constraints to livestock grazing intensity. Global Change Biology.

Fetzel, T., Havlik, P., Herrero, M., Kaplan, J., Kastner, T., Kroisleitner, C., . . . Erb, K.-H. (in review). Massive uncertainties hamper the understanding of global livestock grazing systems. Global Biogeochemical Cycles.

Frankenberg, C., Butz, A., & Toon, G. (2011). Disentangling chlorophyll fluorescence from atmospheric scattering effects in O2 A-band spectra of reflected sun-light. Geophysical Research Letters, 38(3).

Freydank, K., & Siebert, S. (2008). Towards mapping the extent of irrigation in the last century: time series of irrigated area per country. University of Frankfurt (Main), Frankfurt am Main, Germany.

Fyfe, R. M., Woodbridge, J., & Roberts, N. (2015). From forest to farmland: pollen-inferred land cover change across Europe using the pseudobiomiza- tion approach. Global Change Biology, 21(3), 1197–1212.

Giglio, L., Randerson, J. T., & Werf, G. R. (2013). Analysis of daily, monthly, and annual burned area using the fourth-generation global fire emissions database (GFED4). Journal of Geophysical Research: Biogeosciences, 118(1), 317–328.

Guo, K. (2010): Vegetation map and vegetation monographs of China. Bulletin of the Chinese Academy of Sciences, 24.

Haberl, H., Erb, K., Krausmann, F., Gaube, V., Bondeau, A., Plutzar, C., ... Fischer-Kowalski, M. (2007). Quantifying and mapping the human appropriation of net primary production in earth’s terrestrial ecosystems. Proc. Natl. Acad. Sci., 104(31), 12942–12947.

Hengeveld, G. M., Nabuurs, G.-J., Didion, M., van den Wyngaert, I., Clerkx, A., & Schelhaas, M.-J. (2012). A forest management map of European forests. Ecology and Society, 17(4).

Herrero, M., Havlík, P., Valin, H., Notenbaert, A., Rufino, M. C., Thornton, P. K., . . . Obersteiner, M. (2013). Biomass use, production, feed efficiencies, and greenhouse gas emissions from global livestock systems. Proceedings of the National Academy of Sciences, 110(52), 20888–20893.

Hurtt, G., Chini, L., Frolking, S., Betts, R., Feddema, J., Fischer, G., . . . Wang, Y. (2011). Harmonization of land-use scenarios for the period 1500–2100: 600 years of global gridded annual land-use transitions, wood harvest, and resulting secondary lands. Clim. Change, 1–45.

Hurtt, G., et al. (2017). LUH2; https://cmip.ucar.edu/sites/default/files/lumip/luh2 v2f beta readme.pdf.

Hurtt, G., Pacala, S., Moorcroft, P. R., Caspersen, J., Shevliakova, E., Houghton, R., & Moore, B. (2002). Projecting the future of the US carbon sink. Proceedings of the National Academy of Sciences , 99 (3), 1389–1394.

ICID Databases. (2005). Important Data of ICID Member Countries http://www.sancid.org.za/.

Iizumi, T., & Ramankutty, N. (2016). Changes in yield variability of major crops for 1981–2010 explained by climate change. Environmental Research Letters, 11(3), 034003.

Jung, M., Reichstein, M., Margolis, H. A., Cescatti, A., Richardson, A. D., Arain, M. A., . . . others (2011). Global patterns of land-atmosphere fluxes of carbon dioxide, latent heat, and sensible heat derived from eddy covariance, satellite, and meteorological observations. Journal of Geophysical Research: Biogeosciences, 116(G3).

Kattge, J., Diaz, S., Lavorel, S., Prentice, I., Leadley, P., Bönisch, G., . . . others (2011). TRY–a global database of plant traits. Global Change Biology, 17(9), 2905–2935.

Klein Goldewijk, K., & Verburg, P. H. (2013). Uncertainties in global-scale reconstructions of historical land use: an illustration using the HYDE data set. Landscape ecology, 28(5), 861–877.

Klein Goldewijk, K., Beusen, A., Doelman, J., & Stehfest, E. (2016). New anthropogenic land use estimates for the Holocene; HYDE 3.2. Earth Syst. Sci. Data Discuss.

Krausmann, F., Erb, K.-H., Gingrich, S., Haberl, H., Bondeau, A., Gaube, V., . . . Searchinger, T. D. (2013). Global human appropriation of net primary production doubled in the 20th century. Proceedings of the National Academy of Sciences, 110(25), 10324–10329.

Krausmann, F., Erb, K.-H., Gingrich, S., Lauk, C., & Haberl, H. (2008). Global patterns of socioeconomic biomass flows in the year 2000: A compre- hensive assessment of supply, consumption and constraints. Ecological Economics, 65(3), 471–487.

Lauk, C., & Erb, K.-H. (2009). Biomass consumed in anthropogenic vegetation fires: Global patterns and processes. Ecological Economics, 69(2), 301– 309.

Lawrence, D., Hurtt, G., Arneth, A., Brovkin, V., Calvin, K., Jones, A., ... others (2016). The Land Use Model Intercomparison Project (LUMIP) contribution to CMIP6: rationale and experimental design. Geosci. Model Dev.(9), 2973–2998.

Lieth, H. (n.d.). Primary Productivity of the Biosphere. In R. W. H. Lieth (Ed.), (p. 237).

Lin, M., & Huybers, P. (2012). Reckoning wheat yield trends. Environmental Research Letters, 7(2), 024016.

McGrath, M., Luyssaert, S., Meyfroidt, P., Kaplan, J., Bürgi, M., Chen, Y., . . . Valade, A. (2015). Reconstructing European forest management from 1600 to 2010. Biogeosciences, 12(14), 4291–4316.

Mildrexler, D. J., Zhao, M., Heinsch, F. A., & Running, S. W. (2007). A new satellite-based methodology for continental-scale disturbance detection. Ecological Applications, 17(1), 235–250.

Mitchell, B. (2007a). International historical statistics: Africa, Asia and Oceania 1750–2005. London: Palgrave Macmillan.

Mitchell, B. (2007b). International historical statistics: Europe 1750–2005. London: Palgrave Macmillan.

Mitchell, B. (2007c). International historical statistics: the Americas 1750– 2005. London: Palgrave Macmillan.

Monfreda, C., Ramankutty, N., & Foley, J. (2008). Farming the planet: 2. Geographic distribution of crop areas, yields, physiological types, and net primary production in the year 2000. Global Biogeochem. Cycles, 22(1), 1–19.

Moody, E. G., King, M. D., Platnick, S., Schaaf, C. B., & Gao, F. (2005). Spatially complete global spectral surface albedos: Value-added datasets derived from Terra MODIS land products. IEEE Transactions on Geo- science and Remote Sensing, 43(1), 144–158.

Myneni, R., Hoffman, S., Knyazikhin, Y., Privette, J., Glassy, J., Tian, Y., . . . others (2002). Global products of vegetation leaf area and fraction absorbed PAR from year one of MODIS data. Remote Sensing of Environment, 83(1), 214–231.

Naudts, K., Chen, Y., McGrath, M. J., Ryder, J., Valade, A., Otto, J., & Luyssaert, S. (2016). Europes forest management did not mitigate climate warming. Science, 351(6273), 597–600.

Naudts, K., Ryder, J., McGrath, M., Otto, J., Chen, Y., Valade, A., . . . others (2015). A vertically discretised canopy description for ORCHIDEE (SVN r2290) and the modifications to the energy, water and carbon fluxes. Geoscientific Model Development, 8, 2035–2065. Olson, D. M., Dinerstein, E., Wikramanayake, E. D., Burgess, N. D., Powell,

G. V., Underwood, E. C., . . . others (2001). Terrestrial ecoregions of the world: A new map of life on earth: A new global map of terrestrial ecore- gions provides an innovative tool for conserving biodiversity. BioScience, 51(11), 933–938.

Pan, Y., Birdsey, R., Fang, J., Houghton, R., Kauppi, P., Kurz, W., . . . others (2011). A large and persistent carbon sink in the world’s forests. Science, 333(6045), 988–993.

Petit, R. J., Brewer, S., Bord ́acs, S., Burg, K., Cheddadi, R., Coart, E., ... others (2002). Identification of refugia and post-glacial colonisation routes of European white oaks based on chloroplast DNA and fossil pollen evidence. Forest ecology and management, 156(1), 49–74.

Petit, R. J., Hu, F. S., & Dick, C. W. (2008). Forests of the past: a window to future changes. Science, 320(5882), 1450–1452.

Petz, K., Alkemade, R., Bakkenes, M., Schulp, C. J., van der Velde, M., & Leemans, R. (2014). Mapping and modelling trade-offs and synergies between grazing intensity and ecosystem services in rangelands using global-scale datasets and models. Global Environmental Change, 29, 223–234.

Potter, P., Ramankutty, N., Bennett, E. M., & Donner, S. D. (2010). Char- acterizing the spatial patterns of global fertilizer application and manure production. Earth Interactions, 14(2), 1–22.

Poulter, B., Aragao, L., Andela, N., Ciais, P., Kato, T., Lin, X., . . . Shivdenko, A. (in prep.). Mapping global forest age for carbon cycle studies.

Ramankutty, N., Evan, A. T., Monfreda, C., & Foley, J. A. (2008). Farming the planet: 1. Geographic distribution of global agricultural lands in the year 2000. Global Biogeochemical Cycles, 22(1).

Ramankutty, N., & Foley, J. (1999). Estimating historical changes in global land cover: Croplands from 1700 to 1992. Global Biogeochem. Cycles, 13(4), 997–1027.

Ray, D. K., Ramankutty, N., Mueller, N. D., West, P. C., & Foley, J. A. (2012). Recent patterns of crop yield growth and stagnation. Nature Communications, 3, 1293.

Robin, V., Bork, H.-R., Nadeau, M.-J., & Nelle, O. (2014). Fire and forest history of central European low mountain forest sites based on soil charcoal analysis: The case of the eastern Harz. The Holocene, 24(1), 35–47.

Roman-Cuesta, R. M., Rufino, M. C., Herold, M., Butterbach-Bahl, K., Rosen- stock, T. S., Herrero, M., . . . others (2016). Hotspots of gross emissions from the land use sector: patterns, uncertainties, and leading emission sources for the period 2000–2005 in the tropics. Biogeosciences, 13(14), 4253–4269.

Salmon, J. M., Friedl, M. A., Frolking, S., Wisser, D., & Douglas, E. M. (2015). Global rain-fed, irrigated, and paddy croplands: A new high resolution map derived from remote sensing, crop inventories and climate data. International Journal of Applied Earth Observation and Geoinformation, 38, 321–334.

Shevliakova, E., Pacala, S., Malyshev, S., Hurtt, G., Milly, P., Caspersen, J., . . . Crevoisier, C. (2009). Carbon cycling under 300 years of land use change: Importance of the secondary vegetation sink. Global Biogeochem. Cycles, 23(2), GB2022.

Shevliakova, E., Stouffer, R. J., Malyshev, S., Krasting, J. P., Hurtt, G. C., & Pacala, S. W. (2013). Historical warming reduced due to enhanced land carbon uptake. Proceedings of the National Academy of Sciences , 110 (42), 16730–16735.

Siebert, S. (2008). Technical report documentation irrigation 1700–1900. Bonn: University of Bonn.

Siebert, S., Kummu, M., Porkka, M., Döll, P., Ramankutty, N., & Scanlon, B. R. (2015). A global data set of the extent of irrigated land from 1900 to 2005. Hydrology and Earth System Sciences, 19(3), 1521–1545.

Simons, H., Soto, X., Zhu, Z., Singh, K. D., Bellan, M.-F., Iremonger, S., . . . others (2001). Global ecological zoning for the global forest resources assessment 2000 — final report. Food and Agriculture Organization of the United Nations, Forestry Department.

Smith, P., Haberl, H., Popp, A., Erb, K.-h., Lauk, C., Harper, R., ... others (2013). How much land-based greenhouse gas mitigation can be achieved without compromising food security and environmental goals? Global Change Biology, 19(8), 2285–2302.

ter Steege, H., Pitman, N. C., Sabatier, D., Baraloto, C., Salomao, R. P., Guevara, J. E., . . . others (2013). Hyperdominance in the Amazonian tree flora. Science, 342(6156), 1243092.

Tilman, D., Balzer, C., Hill, J., & Befort, B. L. (2011). Global food demand and the sustainable intensification of agriculture. Proceedings of the National Academy of Sciences, 108(50), 20260–20264.

Trondman, A.-K., Gaillard, M.-J., Mazier, F., Sugita, S., Fyfe, R., Nielsen, A. B., . . . others (2015). Pollen-based quantitative reconstructions of Holocene regional vegetation cover (plant-functional types and land-cover types) in Europe suitable for climate modelling. Global Change Biology, 21(2), 676–697.

United States Department of Agriculture (USDA). (2013). Fertilizer use and price; https://www.ers.usda.gov/data-products/fertilizer-use-and-price/.

Van der Werf, G. R., Randerson, J. T., Giglio, L., Collatz, G., Mu, M., Kasibhatla, P. S., . . . van Leeuwen, T. T. (2010). Global fire emissions and the contribution of deforestation, savanna, forest, agricultural, and peat fires (1997–2009). Atmospheric Chemistry and Physics , 10 (23), 11707–11735.

Vilén, T., Gunia, K., Verkerk, P., Seidl, R., Schelhaas, M.-J., Lindner, M., & Bellassen, V. (2012). Reconstructed forest age structure in Europe 1950–2010. Forest Ecology and Management, 286, 203–218.

Wint, W., & Robinson, T. P. (2007). Gridded livestock of the world 2007. Food and Agriculture Organization of the United Nations Rome.

Wirsenius, S. (2003). The biomass metabolism of the food system: A model- based survey of the global and regional turnover of food biomass. Journal of Industrial Ecology, 7(1), 47–80.

Yaroshenko, A. Y., Bartalev, S. A., Ershov, D. V., Isaev, A. S., Potapov, P. V., & Turubanova, S. (2005). Proceedings of the ForestSat conference Operational Tools in Forestry Using Remote Sensing Techniques (Vol. 1). Boras.
